# Crosstalk of regulated cell death pathways in sepsis-associated acute kidney injury: implications for therapy

**DOI:** 10.3389/fcell.2026.1907693

**Published:** 2026-08-24

**Authors:** Xi-Long Liu, Lei Ma

**Affiliations:** 1 Department of Emergency Medical, General Hospital of Ningxia Medical University, Yinchuan, Ningxia, China; 2 School of Clinical Medicine, Ningxia Medical University, Yinchuan, Ningxia, China

**Keywords:** apoptosis, autophagy, ferroptosis, molecular crosstalk, necroptosis, pyroptosis, regulated cell death, sepsis-associated acute kidney injury

## Abstract

Sepsis-associated acute kidney injury (SA-AKI) is a common and severe complication in critically ill patients, independently associated with significantly increased mortality and a high risk of progression to chronic kidney disease. The pathogenesis of SA-AKI is complex and involves not only traditional concepts such as renal hypoperfusion, microcirculatory dysfunction, and direct tubular injury, but also, more importantly, the disordered activation of the host immune response. In recent years, regulated cell death (RCD) has emerged as a central mechanism linking immune dysregulation to tissue damage in SA-AKI. Unlike accidental cell death, RCD is orchestrated by genetically encoded molecular machinery, offering potential targets for therapeutic intervention. This review systematically summarizes the distinct molecular mechanisms of five major RCD pathways, including apoptosis, pyroptosis, necroptosis, autophagy, and ferroptosis, in the pathogenesis of SA-AKI. We highlight the specific contributions of each pathway, their regulatory networks, and the critical molecular players involved. Importantly, we delve into the intricate crosstalk among these pathways, such as caspase-8 acting as a molecular switch between apoptosis, pyroptosis, and necroptosis, the synergistic interplay between Gasdermin D and mixed lineage kinase domain-like protein, the role of autophagy as a “gatekeeper” for other RCDs, the concept of ferritinophagy, and the shared upstream signals including pathogen-associated molecular patterns (PAMPs), damage-associated molecular patterns (DAMPs), Toll-like receptor 4 (TLR4), and oxidative stress. Building upon this mechanistic framework, we further evaluate the current state and future potential of targeted therapeutic strategies aimed at modulating RCD pathways to mitigate renal injury in sepsis. Thus, this review pursues two complementary objectives: to provide a cohesive mechanistic synthesis of RCD crosstalk in SA-AKI, and to propose a theoretical foundation for developing novel, multi-targeted therapies.

## 1 Introduction

Sepsis represents a life-threatening clinical syndrome and remains a leading cause of hospital mortality worldwide. According to the international guidelines for management of sepsis and septic Shock 2026, sepsis is defined as “life-threatening organ dysfunction caused by a dysregulated host response to infection”. Septic shock, a more severe form, requires vasopressors to maintain mean arterial pressure (MAP) ≥65 mmHg together with serum lactate >2 mmol/L (18 mg/dL) in the absence of hypovolemia, and carries a mortality rate exceeding 40% (Cecconi et al., 2018; Prescott et al., 2026). Even in non-shock sepsis, the mortality rate remains approximately 20%–30%, representing a major global health burden despite advances in intensive care and antimicrobial therapy. As the incidence of sepsis continues to rise, increasing attention has been directed toward its complications, notably sepsis-associated acute kidney injury (SA-AKI) (Peerapornratana et al., 2019). Epidemiological data indicate that AKI occurs frequently among septic patients in intensive care units (ICUs), with a pooled incidence of approximately 40%–52% depending on the diagnostic criteria used, and associated fatality rates reaching as high as 48% (Donaldson et al., 2024; Patel et al., 2022). A recent systematic review and meta-analysis of 189 studies involving over 150,000 participants reported that the proportion of septic patients who develop SA-AKI ranges from 26% to 57% depending on the definition applied, with a mortality rate of 48% among those who develop SA-AKI (Donaldson et al., 2024). According to the 2020 Kidney Disease: Improving Global Outcomes (KDIGO) consensus, AKI is defined by any of the following: a serum creatinine increase of ≥50% within 7 days, an absolute increase of ≥0.3 mg/dL within 2 days, or oliguria lasting at least 6 h (Peerapornratana et al., 2019; Goyal et al., 2026). The development of AKI in septic patients markedly worsens prognosis, increasing both short-term mortality and the long-term risk of progressing to chronic kidney disease (CKD) (Peerapornratana et al., 2019).

SA-AKI is characterized by a rapid decline in renal function, with the kidney being particularly vulnerable among all affected organs. The pathogenesis of SA-AKI is complex and multifactorial. Historically, it has been attributed to renal hypoperfusion, microcirculatory dysfunction, and direct tubular injury by inflammatory mediators. However, emerging evidence indicates that the pathophysiology of SA-AKI is not merely a consequence of hemodynamic compromise but rather involves a dynamic interplay between inflammatory dysregulation and metabolic reprogramming. Despite advances in critical care medicine, the precise molecular mechanisms remain incompletely understood, and no targeted therapies are currently available. Existing management is largely supportive, including fluid resuscitation, vasopressors, and renal replacement therapy (Legrand et al., 2024). Cell death is broadly classified into regulated cell death (RCD) and accidental cell death (ACD). Regulated cell death can be further divided into caspase-dependent forms, such as apoptosis and pyroptosis, and caspase-independent forms, including necroptosis, ferroptosis, and autophagy. Accidental cell death, which primarily corresponds to necrosis, is generally considered an unprogrammed process. In contrast, a wide range of programmed cell death modalities have been described, including apoptosis, pyroptosis, necroptosis, ferroptosis, autophagy-dependent cell death, entotic cell death, netotic cell death, parthanatos, lysosome-dependent cell death, alkaliptosis, and oxeiptosis (Tang et al., 2019). In recent years, RCD has emerged as a central mechanistic link between the dysregulated host response and tissue damage in SA-AKI. Unlike accidental cell death, RCD is executed by genetically encoded molecular machinery that offers potential therapeutic targets. Several distinct RCD forms have been implicated in SA-AKI, including pyroptosis, apoptosis, necroptosis, autophagy-dependent cell death, and ferroptosis. Pyroptosis, a pro-inflammatory RCD mode triggered by inflammasome activation, depends on caspase cleavage of Gasdermin D (GSDMD), leading to pore formation and release of IL-1β/IL-18, thereby amplifying renal inflammation (Zhang et al., 2021; Lieberman et al., 2019). However, pyroptosis does not act in isolation. Emerging evidence indicates that other RCD modalities, such as apoptosis, necroptosis, autophagy, and ferroptosis, which also contribute critically to SA-AKI, often through interconnected signaling networks. For instance, caspase-8 functions as a molecular switch orchestrating apoptosis, pyroptosis, or necroptosis depending on cellular context. Autophagy can exert protective or detrimental effects based on its magnitude, and ferroptosis,an iron-dependent, lipid peroxidation-driven cell death, which has recently been recognized as a key player in SA-AKI.

**FIGURE 1:**
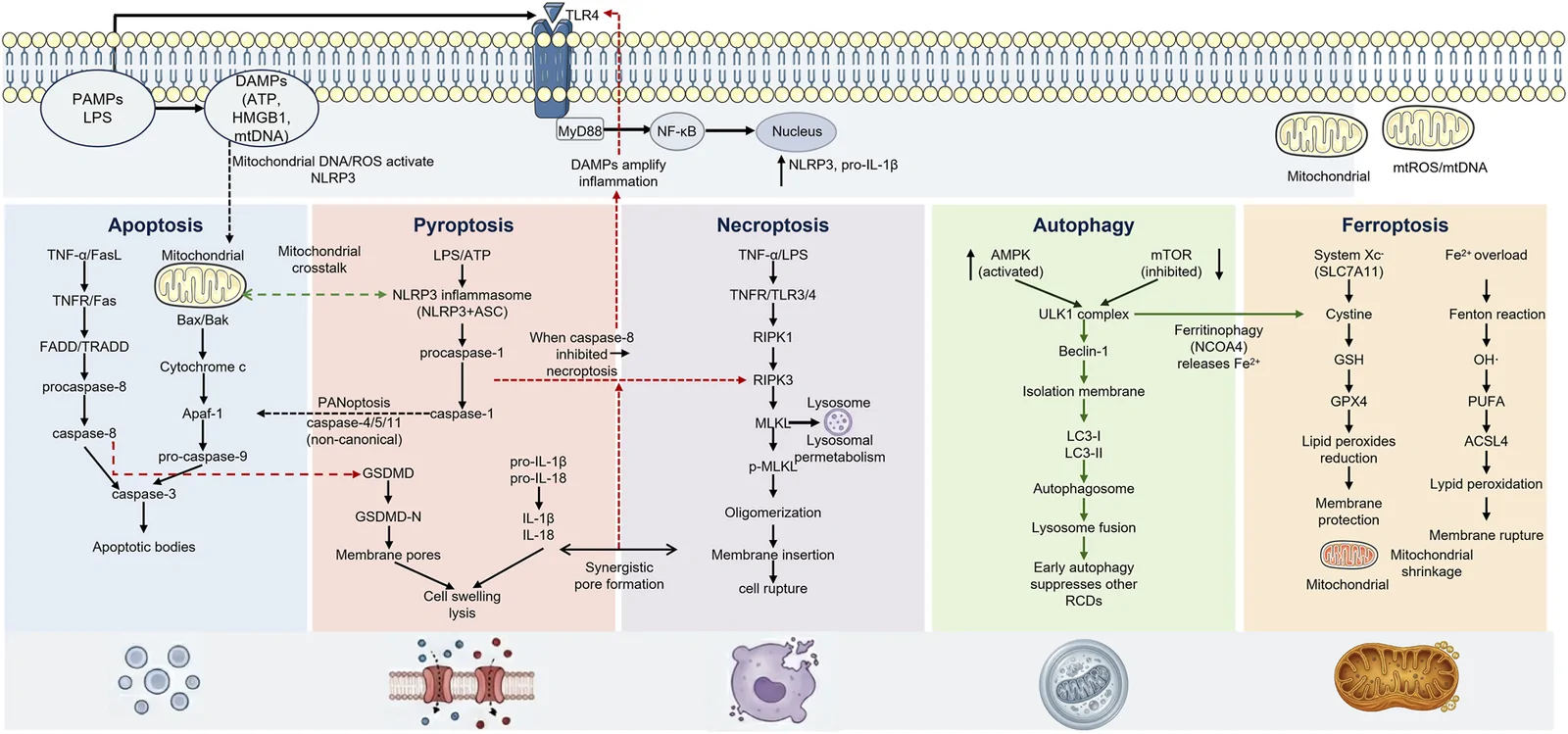
Schematic overview of upstream signaling and interplays among apoptosis (blue), pyroptosis (red), necroptosis (purple), autophagy (green), and ferroptosis (orange) in SA-AKI. Upstream PAMPs/DAMPs trigger TLR4/NF-κB signaling and mitochondrial dysfunction to initiate all RCD cascades. Each column in the middle panel depicts the core molecular cascade of one RCD modality. The bottom panel presents characteristic cellular death phenotypes and corresponding targeted inhibitors. Arrow notation: Solid black arrows: Direct molecular activation and signal propagation. Red dashed arrows: Cell death switching and inflammatory feedback. Double-headed black arrows: Synergistic effects of GSDMD and MLKL, wherein GSDMD-mediated pyroptosis and MLKL-mediated necroptosis converge on membrane pore formation and jointly amplify inflammatory responses. Green solid arrows: Regulatory effect of ferritinophagy on ferroptosis. Green bidirectional dashed arrows: Mitochondrial crosstalk. Black dashed arrows: Indirect activation of NLRP3 by mtDNA/mtROS. Caspase-8 functions as a key molecular switch governing the cell death fate. Autophagy serves as a dynamic gatekeeper that limits excessive RCD activation, while ferritinophagy links autophagy to ferroptosis. These interconnected pathways collectively contribute to renal damage in sepsis.

Previous reviews have discussed individual RCD pathways in SA-AKI, and their contributions are duly acknowledged. However, a comprehensive synthesis that simultaneously integrates the mechanistic roles of all five major RCD types within a unified framework remains lacking. Notably, classical apoptosis, despite being the best-characterized form of programmed cell death, has not been fully incorporated into the current RCD framework of SA-AKI. Moreover, crosstalk among different RCD pathways, such as the role of caspase-8 as a molecular switch among apoptosis, pyroptosis, and necroptosis, and the connection between autophagy and ferroptosis *via* ferritinophagy, has not been comprehensively analyzed in this disease context. Furthermore, the possibility of co-targeting multiple RCD modules as a therapeutic strategy has received limited attention. In this review, we aim to address these gaps. We systematically examine the molecular mechanisms of five principal RCD pathways in SA-AKI pathogenesis, with particular emphasis on their regulatory networks and inter-pathway crosstalk. Based on this mechanistic integration, we then propose a theoretical framework for the development of multi-targeted therapeutic strategies. We also discuss the current challenges and future directions for translating these mechanistic insights into clinical practice **(** Figure 1).

For this review, a comprehensive literature search was conducted in PubMed, Web of Science, and Scopus databases. The search covered publications from January 2000 to December 2025, using the following keywords and their combinations: “sepsis-associated acute kidney injury”, “SA-AKI”, “regulated cell death”, “apoptosis”, “pyroptosis”, “necroptosis”, “autophagy”, “ferroptosis”, “caspase-8”, “GSDMD”, “MLKL”, “ferritinophagy”, “NLRP3 inflammasome”, “RIPK1”, “RIPK3”, and “GPX4”. Only peer-reviewed original articles and review articles published in English were included. Conference abstracts, case reports, and non-English articles were excluded. Priority was given to studies with mechanistic insights, *in vivo* or *in vitro* evidence relevant to SA-AKI, and publications within the last decade. The reference lists of retrieved articles were also manually screened to identify additional relevant studies. Classic foundational studies published before 2000 were included where they provided essential conceptual frameworks or definitive mechanistic descriptions that have not been superseded by more recent work.

## 2 Apoptosis in SA-AKI

Apoptosis is a regulated cell death mechanism and serves as the primary process for eliminating senescent or dysfunctional cells (Tang et al., 2019). Sepsis also induces extensive apoptosis in lymphocytes and dendritic cells, thereby altering immune responses and impairing microbial clearance. Apoptosis is a major characteristic of immune system dysfunction, a feature consistently observed in both human sepsis and experimental sepsis models (Ward, 2008). Consequently, apoptosis is considered one of the widely discussed molecular mechanisms underlying SA-AKI (Fernandes et al., 2008). It has been clearly demonstrated in animal models of sepsis that targeted intervention in apoptosis significantly prolongs the survival time of rats (Aziz et al., 2014). Moreover, inhibition of apoptosis reduces organ dysfunction and mortality (Marshall and Watson, 1997; Coopersmith et al., 2002). Based on the above, we will systematically and specifically elucidate the occurrence of apoptosis in SA-AKI by focusing on the classical apoptotic pathways and key apoptosis-related proteins.

### 2.1 The apoptotic pathway in SA-AKI

Current studies indicate that three major apoptotic pathways are involved in the pathogenesis of SA-AKI. The first is the intrinsic (mitochondrial) apoptotic cascade. In SA-AKI, the intrinsic apoptotic pathway is primarily triggered by excessive reactive oxygen species (ROS) production, inflammatory factors, endoplasmic reticulum (ER) stress, and energy metabolism disturbances. These signals lead to a reduction in the mitochondrial membrane potential of renal tubular epithelial cells (TECs), opening of the permeability transition pore, and activation of the pro-apoptotic proteins Bax/Bak, which form pores in the outer mitochondrial membrane, resulting in mitochondrial outer membrane permeabilization (MOMP). Subsequently, cytochrome c and other pro-apoptotic factors are released into the cytosol. Cytochrome c assembles with Apaf-1 and procaspase-9 to form the apoptosome, which activates caspase-9. Caspase-9 then activates the executioner caspase-3, which cleaves cytoskeletal proteins and DNA, ultimately leading to cell shrinkage, nuclear fragmentation, and the formation of apoptotic bodies that are cleared by phagocytes without eliciting an inflammatory response. During this process, downregulation of the anti-apoptotic proteins Bcl-2/Bcl-xL, upregulation of the pro-apoptotic proteins Puma/Bax, and activation of p53 all promote apoptosis, accelerating renal cell death during sepsis (Su et al., 2023; Song C. et al., 2024; Du et al., 2019; Sun et al., 2021). Chen et al. found that resveratrol inhibits the expression of iNOS, Bcl-2, and Bcl-xL in macrophages, suppresses apoptosis, and thereby alleviates septic kidney injury (Chen et al., 2015). In contrast, p53 deacetylation inhibits apoptosis (Sun et al., 2021). An et al. demonstrated both *in vivo* and *in vitro* that downregulation of Sirtuin 3 (SIRT3) leads to hyperacetylation and inactivation of pyruvate dehydrogenase E1 subunit alpha (PDHA1), resulting in excessive lactate production in renal TECs. Lactate then mediates the lactylation of Fis1 at lysine 20 (Fis1 K20La) (An et al., 2023). Increased Fis1 K20La causes excessive mitochondrial fission, followed by ATP depletion, overproduction of mitochondrial ROS (mtROS), and mitochondrial apoptosis (An et al., 2023). Moderate apoptosis helps eliminate damaged cells and prevent the spread of inflammation, whereas excessive apoptosis leads to a substantial loss of functional tubular units, promoting renal function deterioration and even progression to chronic kidney disease.

The second pathway is the extrinsic apoptotic cascade. In SA-AKI, the extrinsic apoptotic pathway is mainly mediated by death receptors (Li et al., 2021). Specifically, during sepsis, large amounts of death ligands (such as TNF-α and FasL) are released and bind to their corresponding death receptors (e.g., TNFR1 and Fas) on the surface of renal TECs. This induces receptor trimerization and the recruitment of adaptor proteins (e.g., TRADD and FADD) and procaspase-8, forming the death-inducing signaling complex (DISC). Within the DISC, procaspase-8 is autocatalytically cleaved to generate active caspase-8. Activated caspase-8 directly cleaves and activates the executioner caspase-3, which then cleaves cytoskeletal proteins and DNA repair enzymes, ultimately leading to cell shrinkage, nuclear fragmentation, and the formation of apoptotic bodies that are cleared by phagocytes without causing significant inflammation. In addition, in certain cell types, caspase-8 can cleave the Bid protein to produce truncated tBid, which translocates to mitochondria and amplifies the intrinsic apoptotic pathway. Excessive activation of the extrinsic pathway in SA-AKI aggravates the loss of renal TECs and renal dysfunction, whereas inhibition of caspase-8 or blockade of death receptor signaling attenuates kidney injury (Miao et al., 2020; Zheng et al., 2023a). Bircan et al. found that capsaicin inhibits caspase-8, reduces inflammation, oxidative damage, and apoptosis, and exerts a renoprotective effect in sepsis-induced acute kidney injury (Bircan et al., 2025).

The third pathway is the ER stress-mediated apoptotic pathway. In SA-AKI, activation of the ER apoptotic pathway is mainly attributed to sepsis-induced hypoxia, oxidative stress, calcium homeostasis imbalance, and energy metabolism disturbances. These stimuli cause extensive accumulation of unfolded or misfolded proteins in the ER of renal TECs, triggering ER stress. ER stress initiates the unfolded protein response through three sensors: PERK, IRE1α, and ATF6. However, under persistent or excessive stress, the PERK pathway upregulates the transcription factor CHOP, which inhibits the anti-apoptotic protein Bcl-2, upregulates the pro-apoptotic proteins Bax/Bak, and simultaneously activates ER-resident caspase-12 (in rodents) or caspase-4 (in humans). Activated caspase-12/caspase-4 further activates caspase-9, which in turn activates executioner caspase-3, ultimately leading to cytoskeletal breakdown, DNA damage, and apoptosis (Zhou et al., 2025). Moreover, the IRE1α pathway can activate JNK signaling by recruiting TRAF2 and ASK1, and JNK can also promote mitochondrial-mediated apoptosis (Bai G. et al., 2022). The ER stress and mitochondrial apoptotic pathways intersect, together exacerbating the loss of renal TECs and renal function impairment in SA-AKI. Using an LPS-induced model of septic kidney injury, Ebselen was found to alleviate ER stress, inhibit apoptosis, and ameliorate SA-AKI by modulating the expression of eukaryotic translation initiation factor 2-alpha kinase 3 (EIF2AK3) and GRP78 in kidney tissue, as well as activating transcription factor 4 (ATF4) and activating transcription factor 6 (ATF6) in the serum (Karaboğa et al., 2025).

### 2.2 Key molecules in SA-AKI

#### 2.2.1 The key molecule of the intrinsic pathway: Bax

Bax is a core pro-apoptotic molecule of the intrinsic (mitochondrial) apoptotic pathway. Under physiological conditions, Bax exists as an inactive monomer in the cytoplasm of renal TECs. During sepsis, signals such as oxidative stress, ER stress, and DNA damage activate p53 and upregulate Bax expression, while simultaneously downregulating the anti-apoptotic proteins Bcl-2/Bcl-xL, thereby disturbing the Bcl-2 family balance (Peña-Blanco and Bax, 2018). Activated Bax undergoes a conformational change and translocates to the outer mitochondrial membrane, where it oligomerizes to form transmembrane pores, leading to MOMP and the release of cytochrome c and Smac/DIABLO into the cytosol (Spitz and Gavathiotis, 2022). Released cytochrome c assembles with Apaf-1 and procaspase-9 to form the apoptosome, activating caspase-9 and subsequently executioner caspase-3, ultimately inducing apoptosis of renal TECs. Studies have shown that the Bax/Bcl-2 expression ratio is increased in SA-AKI, and reducing this ratio suppresses apoptosis in renal TECs (Luo et al., 2016). Gu et al. demonstrated in an LPS-induced sepsis model in C57BL/6 J mice and HK-2 cells that magnesium sulfate (MgSO_4_) reduces the Bax/Bcl-2 ratio both *in vitro* and *in vivo*, thereby inhibiting intrinsic apoptosis, alleviating oxidative stress and mitochondrial dysfunction in HK-2 cells, decreasing mitochondrial calcium load and ROS production, and restoring ATP content, antioxidant capacity, and mitochondrial membrane potential, ultimately improving SA-AKI (Gu et al., 2026). These findings confirm that Bax is a critical effector molecule in this pathway.

#### 2.2.2 The key molecule of the extrinsic pathway: caspase-8

Caspase-8 is the initiator caspase of the extrinsic apoptotic pathway. In SA-AKI, large amounts of inflammatory cytokines (especially TNF-α and FasL) induced by sepsis bind to the death receptors TNFR1 and Fas on the surface of renal TECs, recruiting the adaptor proteins FADD and procaspase-8 to form the DISC. Within the DISC, procaspase-8 is autocatalytically cleaved to active caspase-8. Activated caspase-8 directly cleaves and activates executioner caspase-3, inducing apoptosis (Liu D. et al., 2023); it can also cleave Bid to produce truncated tBid, which translocates to mitochondria and triggers the intrinsic pathway, thereby amplifying the apoptotic signal (Pahlavani, 2022). Furthermore, the activity status of caspase-8 determines the mode of cell death: when caspase-8 is inhibited, cells tend to undergo necroptosis (*via* the RIPK1/RIPK3/MLKL pathway), thus caspase-8 is regarded as a molecular switch between apoptosis and necroptosis (Speir et al., 2020). Studies have shown that RIPK1, IL-1β, and IL-18 levels are positively correlated with the length of ICU stay and disease severity, whereas caspase-8 levels are negatively correlated (Briassoulis et al., 2025). In SA-AKI models, inhibition of caspase-8 activity significantly reduces renal TEC apoptosis and ameliorates renal dysfunction (Miao et al., 2020).

#### 2.2.3 The key molecule of the ER stress-mediated apoptotic pathway: CHOP

CHOP (C/EBP homologous protein) is a key transcription factor in ER stress-induced apoptosis. During sepsis, renal TECs undergo ER stress due to hypoxia, ROS accumulation, and calcium homeostasis imbalance, activating the three sensing pathways: PERK, IRE1α, and ATF6. Among these, the PERK pathway selectively upregulates CHOP expression *via* phosphorylation of eIF2α (Kong et al., 2022). Once translocated into the nucleus, CHOP promotes apoptosis through multiple mechanisms: (1) inhibiting the transcription of the anti-apoptotic protein Bcl-2; (2) upregulating the expression of the pro-apoptotic proteins Bax and Bak; and (3) activating ER-resident caspase-12 (caspase-4 in humans), which then activates caspase-9 and caspase-3 (Farahani et al., 2024). Downregulation of glucose-regulated protein 78 (GRP78) affects CHOP expression, inhibiting immune response/oxidative stress-related apoptosis and alleviating LPS-induced acute kidney injury (Teng et al., 2018). Chen et al. established a sepsis mouse model using LPS and found that LPS administration induced a strong ER stress response in the kidney, characterized by upregulation of GRP78, activation of PERK, phosphorylation of eIF2α, and overexpression of CHOP; these responses were significantly inhibited by pretreatment with FTA (Chen Y. et al., 2023). Therefore, the CHOP-mediated ER stress apoptotic pathway plays a crucial role in SA-AKI.

## 3 Pyroptosis in SA-AKI

Pyroptosis is a form of programmed cell death characterized by a strong inflammatory response and is closely associated with the development and progression of sepsis and its related organ injuries. Unlike the non-inflammatory clearance mechanism of apoptosis, pyroptosis manifests as cell swelling, formation of plasma membrane pores, membrane rupture, and the release of large amounts of pro-inflammatory cytokines (e.g., IL-1β and IL-18), thereby exacerbating local and systemic inflammatory cascades (Ding et al., 2016). In SA-AKI, pyroptosis has been identified as a critical mechanism leading to the death of renal TECs and the deterioration of renal function (Miao et al., 2010). Li et al. found that NLRP3 expression was upregulated and the inflammatory response was enhanced in renal tissues of CLP-induced septic mice (Li et al., 2022a). Numerous studies have demonstrated that inhibiting key pyroptosis molecules (such as the NLRP3 inflammasome, caspase-1/11, and GSDMD) significantly alleviates sepsis-associated kidney injury (Xu et al., 2024). In this section, we systematically describe the canonical pyroptosis pathways (caspase-1-dependent and caspase-1-independent) in SA-AKI and focus on the specific mechanisms of key molecules (NLRP3 inflammasome, GSDMD, and IL-1β/IL-18) in renal injury, thereby providing a theoretical basis for targeting pyroptosis in SA-AKI therapy.

### 3.1 The pyroptotic pathway in SA-AKI

The pyroptosis involved in SA-AKI pathogenesis primarily relies on inflammasome activation, which engages certain caspase family members to cleave and activate gasdermin proteins. The activated gasdermin proteins then translocate to the membrane, form pores, cause cell swelling and cytoplasmic leakage, and eventually lead to membrane rupture and pyroptotic cell death. Based on caspase dependency, this process is divided into two classical pathways: the caspase-1-dependent pyroptosis pathway and the caspase-1-independent pyroptosis pathway. In SA-AKI, the canonical (caspase-1-dependent) pyroptosis pathway is initiated when pattern recognition receptors (e.g., NLRP3) in renal TECs or immune cells recognize pathogen-associated molecular patterns (PAMPs) or damage-associated molecular patterns (DAMPs). Subsequently, the receptor recruits procaspase-1 *via* the adaptor protein ASC, assembling into an inflammasome that promotes autocatalytic cleavage of procaspase-1 into active caspase-1. Activated caspase-1 cleaves GSDMD to release its N-terminal domain, which oligomerizes in the cell membrane to form pores, leading to cell swelling and membrane rupture. Simultaneously, caspase-1 cleaves pro-IL-1β and pro-IL-18 into their mature forms, which are released through the pores, triggering a robust inflammatory response and ultimately causing pyroptosis of renal TECs (Liu et al., 2016). The non-canonical pyroptosis pathway does not depend on caspase-1. Instead, human caspase-4/5 or mouse caspase-11 directly recognize intracellular lipopolysaccharide (LPS), dimerize, and become activated. These activated caspases also cleave GSDMD to form membrane pores and induce pyroptosis. In addition, they can indirectly activate the NLRP3 inflammasome by triggering potassium efflux and activating the Pannexin-1 channel, which in turn activates caspase-1 and further amplifies the inflammatory response. Both pathways converge on GSDMD pore formation as the common terminal step, synergistically exacerbating tubular injury and the inflammatory cascade in SA-AKI.

### 3.2 Key molecules in SA-AKI

Analogous to its role in other pathophysiological processes, pyroptosis in SA-AKI involves key molecules including GSDMD, the NLRP3 inflammasome, caspase family proteases, and the downstream pro-inflammatory cytokines IL-1β and IL-18. In addition, upstream regulatory molecules such as ZBP1, NF-κB, and mitochondrial reactive oxygen species (mtROS) also play significant roles.

#### 3.2.1 GSDMD: the final executor of pyroptosis

GSDMD is a member of the gasdermin family. Its protein structure contains a cytotoxic N-terminal domain and a C-terminal inhibitory domain connected by a flexible linker. In the resting state, the C-terminal domain inhibits the pore-forming activity of the N-terminus. Upon activation of pyroptotic signals, caspase-1 or caspase-11 (human caspase-4/5) specifically cleave GSDMD at the interdomain linker, releasing an approximately 31 kDa N-terminal fragment (GSDMD-N) (Shi et al., 2015; Shi et al., 2017). This fragment rapidly translocates to the inner leaflet of the plasma membrane, interacts with membrane lipids, and oligomerizes. It first forms a non-inserted “pre-pore” structure, then undergoes conformational rearrangement to form crescent-shaped oligomers, and finally assembles into annular membrane pores with an inner diameter of 10–15 nm (Deng J. et al., 2021; Dubyak et al., 2023). These pores not only cause osmotic swelling and lysis of the cell but also allow passive release of mature IL-1β and IL-18 (approximately 17–18 kDa) into the extracellular space (Barnett and Ting, 2020; Xiaodong and Xuejun, 2023). Yang et al. found that GSDMD−/− mice exhibited attenuated organ injury compared to wild-type mice in a sepsis model (Yang W. et al., 2024). Animal experiments have shown that GSDMD gene knockout or pharmacological inhibition significantly reduces renal TEC injury, lowers serum creatinine and blood urea nitrogen levels, and improves survival rates in septic mice (Silva et al., 2021; Kang et al., 2018) Furthermore, inhibiting GSDMD can block the formation of neutrophil extracellular traps (NETs), thereby mitigating multiple organ dysfunction (Silva et al., 2021).

#### 3.2.2 The NLRP3 inflammasome: the initiation platform for pyroptosis in SA-AKI

The NLRP3 inflammasome is a well-studied pyroptosis-initiating complex. It is a large multiprotein complex with a molecular weight of approximately 700 kDa, composed of NLRP3, the adaptor protein ASC, and the effector protein caspase-1 (Lamkanfi et al., 2014; Zhao H. et al., 2018) Assembly of the NLRP3 inflammasome requires interactions among the NLRP3 receptor, the ASC adaptor, and pro-caspase-1. Under normal physiological conditions, NLRP3 is maintained in an auto-inhibited state. When the cell senses PAMPs (e.g., LPS) or DAMPs (e.g., ATP, uric acid, mtDNA), NLRP3 undergoes a conformational change. Its N-terminal PYD domain recruits the adaptor protein ASC, which also contains a PYD domain; the CARD domain of ASC then recruits pro-caspase-1, completing inflammasome assembly. ASC is predominantly localized in the nucleus of human monocytes/macrophages but rapidly translocates to the cytoplasm under stress, linking NLRP3 to pro-caspase-1 and promoting NLRP3 inflammasome activation. Caspase-1, also known as IL-1β converting enzyme, is the effector protein of the NLRP3 inflammasome. It is activated by autocatalytic cleavage of its precursor, pro-caspase-1, to form active caspase-1, thereby generating the functional inflammasome complex (Kelley et al., 2019; Huang et al., 2021). Studies have shown that the expression levels of NLRP3, ASC, and caspase-1 are significantly elevated in renal tissues of SA-AKI patients and animal models, and these levels are positively correlated with the degree of tubular injury (Wang et al., 2021; Li et al., 2022b; Lin et al., 2021). Upon activation, the NLRP3 inflammasome directly cleaves GSDMD to induce pyroptosis and also promotes the maturation and secretion of IL-1β and IL-18 (Lamkanfi et al., 2014; Zhao H. et al., 2018; Yang et al., 2019). It is noteworthy that activation of the NLRP3 inflammasome is tightly regulated by two signals: the first signal, *via* the NF-κB pathway, upregulates the expression of NLRP3 and pro-IL-1β; the second signal is triggered by various NLRP3 agonists (e.g., K^+^ efflux, mtROS, lysosomal rupture), which induce complex assembly (Lamkanfi et al., 2014; Yang et al., 2019). In SA-AKI models established by LPS injection in mice or LPS treatment of HK-2 cells *in vitro*, activation of the NLRP3 inflammasome induced renal cell pyroptosis, and indirect regulation of pyroptosis by the NLRP3 inflammasome was mediated by caspase-1, ROS, and Panx1 signaling pathways (Huang et al., 2020). Furthermore, that study demonstrated that Panx1 inhibition reduced NLRP3 inflammasome activation, decreased the production of IL-1β, IL-6, and TNF-α in SA-AKI, and ultimately alleviated septic kidney injury (Huang et al., 2020).

#### 3.2.3 Caspase family: the catalytic core of pyroptosis in SA-AKI

The caspase family plays a central catalytic role in pyroptosis and is divided into canonical and non-canonical pathways (Shi et al., 2017; Bu et al., 2021). The **canonical pathway** is primarily mediated by caspase-1. Activated caspase-1 not only cleaves GSDMD but also cleaves inactive pro-IL-1β and pro-IL-18 into their mature forms, thereby simultaneously executing cell death and amplifying inflammation. The non-canonical pathway is mediated by mouse caspase-11 and its human homologs caspase-4/5. These caspases can directly recognize intracellular LPS from Gram-negative bacteria and cleave GSDMD independently of upstream inflammasomes, inducing pyroptosis (Bu et al., 2021; Zhai et al., 2023). Studies have shown that in LPS-induced SA-AKI mouse models, knockout of caspase-1 or caspase-11 significantly reduces pyroptosis of renal TECs, lowers inflammatory factor levels, and improves renal function (Ye et al., 2019; Yang et al., 2022). In addition, VX-765, a selective caspase-1 inhibitor, has demonstrated renoprotective effects in models of diabetic nephropathy and sepsis, reducing the maturation and release of IL-1β (Song et al., 2018; Wen et al., 2022).

#### 3.2.4 Pro-inflammatory cytokines: effector molecules of pyroptosis in SA-AKI

IL-1β and IL-18 are the main pro-inflammatory cytokines released during pyroptosis (Xiong et al., 2020; Fujimura et al., 2023). Both are present in the cytoplasm as inactive precursors (pro-IL-1β and pro-IL-18). After being cleaved by caspase-1, they become mature molecules and are released extracellularly through GSDMD pores (Exconde et al., 2023; Shi et al., 2023). In SA-AKI, elevated IL-1β and IL-18 act on glomerular endothelial cells and peritubular capillary endothelial cells, inducing endothelial dysfunction, increased vascular permeability, leukocyte adhesion and infiltration, and glycocalyx degradation, ultimately leading to microcirculatory flow disturbances and tubular ischemic injury (Kelley et al., 2019; Tang et al., 2024). Studies have shown that endothelial dysfunction caused by inflammatory and oxidative mediators, along with coagulation disorders and glycocalyx disruption, can lead to microcirculatory dysfunction even when macrovascular status is preserved (Peerapornratana et al., 2019; Zafrani et al., 2015; Gotts and Matthay, 2016). Functional studies using intravital video microscopy in LPS-induced septic mice demonstrated impaired peritubular capillary blood flow, resulting in tubular stress and renal injury (Wu et al., 2007). Clinical studies have revealed that plasma IL-18 levels in septic patients are significantly correlated with AKI severity and poor prognosis (Herman and Pasinetti, 2018; Krishnan et al., 2014). Furthermore, the IL-1β receptor antagonist Anakinra has shown potential in reducing inflammation and organ injury in some sepsis clinical trials (Rajasekaran et al., 2014; Pacini et al., 2018).

#### 3.2.5 Other molecules involved in pyroptosis in SA-AKI

ZBP1 (Z-DNA binding protein 1) is a recently identified key upstream regulator of pyroptosis. As an intracellular nucleic acid sensor, ZBP1 recognizes Z-form nucleic acids of pathogenic or endogenous origin (e.g., mitochondrial DNA) and subsequently assembles the PANoptosome complex, which integrates the three signaling pathways of pyroptosis, apoptosis, and necroptosis (Malireddi et al., 2019; Qi et al., 2023; Song Q. et al., 2024). ZBP1 recruits RIPK3 and caspase-8, activates the NLRP3 inflammasome and caspase-1, and thereby executes pyroptosis (Lee et al., 2021). In sepsis models, ZBP1 deficiency in macrophages reduced mitochondrial damage, inhibited glycolysis, attenuated NLRP3-mediated pyroptosis, and improved endothelial dysfunction (Gong et al., 2024). In addition, the NF-κB signaling pathway plays a central role in the “priming” phase of pyroptosis. Upon LPS recognition by Toll-like receptors (e.g., TLR4), NF-κB is activated *via* the MyD88-dependent pathway, leading to upregulated transcription of NLRP3, pro-IL-1β, and pro-IL-18 (Li W. et al., 2022; De Souza et al., 2012). In SA-AKI animal models, Resolvin D1 and β-Casomorphin-7 significantly reduced the production of inflammatory factors in renal tissue and alleviated renal TEC pyroptosis by inhibiting the NF-κB pathway (Wang et al., 2022; Zhang Z. et al., 2019). Finally, mitochondrial reactive oxygen species (mtROS) and mitochondrial DNA (mtDNA), as important DAMPs, are produced in large quantities in sepsis-induced renal tubular cells. MtROS directly activate the NLRP3 inflammasome, while oxidized mtDNA released into the cytosol further enhances inflammasome signaling (Su et al., 2023; Zhang et al., 2022). Studies have shown that antioxidants such as MitoQ and NAC, or mitophagy enhancers, can reduce mtROS, inhibit NLRP3 activation, and thereby alleviate SA-AKI in septic animal or cell models (Lin et al., 2019; Xia et al., 2019; Fan et al., 2020).

In SA-AKI, the canonical pyroptosis pathway is primarily initiated by the NLRP3 inflammasome, followed by caspase-1-mediated cleavage of GSDMD to form membrane pores, while simultaneously promoting the maturation and release of IL-1β and IL-18, ultimately leading to inflammatory death of renal TECs and renal dysfunction. Upstream regulatory factors such as ZBP1, NF-κB, and mitochondrial oxidative stress amplify the pyroptotic response. These key molecules and their interacting pathways constitute the core molecular network of pyroptosis in SA-AKI, providing important targets for the development of novel therapeutic strategies.

## 4 Autophagy in SA-AKI

Autophagy is a process in which eukaryotic cells utilize lysosomes to degrade their own cytoplasmic proteins and damaged organelles under the regulation of autophagy-related genes (Atgs) (Esrefoglu, 2024). Currently, the role of autophagy in SA-AKI is not fully unified and may present a complex picture in which protective and detrimental effects coexist (Ch et al., 2013; Choi, 2020). On the one hand, studies indicate that autophagy activation alleviates SA-AKI. In animal models of SA-AKI induced by LPS or CLP, the expression of the autophagy-related proteins LC3-II and Beclin-1 is increased, and autophagic flux is enhanced (Wu et al., 2019; Zhao W. et al., 2018; Sang et al., 2021). Leventhal et al. showed that knockout of the *Atg7* gene, which blocks autophagy, resulted in aggravated tubular injury, elevated blood urea nitrogen, and worsened renal function (Leventhal et al., 2016). These findings support a protective role of autophagy in SA-AKI. On the other hand, excessive or dysregulated autophagy may also exacerbate kidney injury. It has been reported that treatment with the autophagy inhibitor 3-MA in a CLP sepsis model reduced the expression of Beclin-1 and the LC3-II/I ratio but unexpectedly alleviated renal damage, suggesting that autophagy may promote pathological processes under certain conditions (Wu et al., 2019). Moreover, autophagy exhibits dynamic changes during different stages of sepsis: it is activated in the early phase to cope with stress, but may become impaired in the late phase due to lysosomal dysfunction, leading to blocked autophagic flux and subsequently aggravated tubular injury (Yin et al., 2016; Ho et al., 2016; Hsiao et al., 2012). Current basic research tends to consider that autophagy dysregulation, rather than simply activation or inhibition, is the core cause of pathological damage in SA-AKI (Liu and Chen, 2024; Bhatia and Choi, 2020). Therefore, a deeper understanding of the precise regulatory mechanisms of autophagy in SA-AKI and clarification of the conditions that switch it from protective to detrimental are of great significance for targeting autophagy in the treatment of SA-AKI.

The critical question, therefore, is not merely whether autophagy is protective or detrimental in SA-AKI, but rather what factors determine the switch between these two outcomes within the septic kidney. Several determinants have been identified in SA-AKI models. Cell type specificity represents a primary factor. In renal tubular epithelial cells, autophagy is predominantly cytoprotective during sepsis, as genetic or pharmacological impairment of autophagy in TECs aggravates tubular injury and worsens renal function in LPS-induced SA-AKI models (Leventhal et al., 2016; Bhatia and Choi, 2020). In renal endothelial cells, autophagy also appears to be protective (Li Z.-L. et al., 2024). However, in immune cells such as macrophages, the role of autophagy may be more complex—dysregulated autophagy in these cells can modulate inflammatory responses in ways that are not uniformly beneficial (Liu et al., 2026). Disease stage is a second critical determinant. During the early phase of sepsis, autophagy is rapidly activated to cope with acute stress (Wu Z. et al., 2022), whereas in the late phase, autophagic flux may become impaired (Dai et al., 2019). This temporal pattern suggests that the same pathway can be protective early but detrimental when dysregulated later. The extent of autophagic flux impairment is a third factor: moderate autophagy activation facilitates cellular adaptation and survival, but excessive or blocked autophagy can precipitate autophagic cell death or sensitize cells to other RCD modalities (Ch et al., 2013; Yin et al., 2016; Ho et al., 2016). When autophagosome clearance is compromised due to lysosomal dysfunction, the accumulation of autophagic substrates such as p62 can trigger inflammatory responses and amplify tissue damage (Liu J.-X. et al., 2020; Sunahara et al., 2018; Jin C. et al., 2023). These observations collectively indicate that the functional outcome of autophagy in SA-AKI is context-dependent, shaped by cell type, temporal dynamics, and the integrity of autophagic flux rather than by a simple on-off switch.

### 4.1 Canonical autophagy pathway in SA-AKI

In SA-AKI, renal cells, especially renal tubular epithelial cells (TECs), face multiple stressors including ischemia-hypoxia, lipopolysaccharide (LPS), and inflammatory cytokines. As an important cellular protective mechanism, autophagy is rapidly activated (Wu et al., 2019; Zhao W. et al., 2018; Sang et al., 2021). The canonical autophagy pathway in SA-AKI is primarily regulated by two energy sensors: mammalian target of rapamycin (mTOR) and AMP-activated protein kinase (AMPK). Under physiological conditions, mTOR is active and suppresses autophagy (Li et al., 2017). During SA-AKI, due to energy depletion and oxidative stress, mTOR activity is inhibited, thereby relieving the negative regulation of autophagy (Li et al., 2017; Hsieh et al., 2011). Specifically, when mTOR is inactivated, ULK1 (unc-51-like kinase 1) is dephosphorylated and assembles with FIP200 and Atg13 to form the ULK1 complex, which subsequently activates downstream Beclin-1, initiating autophagy (Ch et al., 2013; Klionsky et al., 2021). Beclin-1, a key molecule in autophagy initiation, is expressed at elevated levels in the renal tissues of SA-AKI patients and animal models, and its levels are positively correlated with autophagic activity (Deng Z. et al., 2021; Bai X. et al., 2022).

Meanwhile, AMPK, as a core sensor of cellular energy metabolism, is activated by ATP depletion during SA-AKI. Activated AMPK not only directly phosphorylates ULK1 at Ser317 and Ser777 to initiate autophagy (Li K. et al., 2019), but also further amplifies autophagic signaling by negatively regulating mTOR (Hsieh et al., 2011). Studies have shown that in LPS-induced AKI models, activation of the AMPK/mTOR pathway significantly enhances autophagy and reduces tubular injury, whereas inhibition of AMPK blocks autophagy and worsens renal dysfunction (Li et al., 2017; Hsieh et al., 2011). These findings establish the AMPK/mTOR signaling axis as one of the core pathways for autophagy activation in SA-AKI.

During the autophagosome formation stage, microtubule-associated protein one light chain 3 (LC3) plays a critical role. LC3-I is conjugated to phosphatidylethanolamine (PE) through the action of Atg4, Atg7, and Atg3, converting into the membrane-bound form LC3-II, which is integrated into the autophagosome membrane (Jiang et al., 2025). The LC3-II/LC3-I ratio is significantly elevated in the renal tissues of SA-AKI animal models and is widely used as a marker of autophagic activity (Sunahara et al., 2018; Jin C. et al., 2023). After formation, autophagosomes fuse with lysosomes to degrade damaged mitochondria, misfolded proteins, and inflammasome complexes, thereby limiting inflammatory injury and apoptosis of renal TECs (Leventhal et al., 2016; Yu et al., 2018). Mice with *Atg7* knockout (which blocks autophagy) exhibited more severe tubular damage and elevated blood urea nitrogen upon LPS stimulation, directly demonstrating the protective role of the canonical autophagy pathway in SA-AKI (Leventhal et al., 2016).

In addition, the NF-κB signaling pathway, as a bridge between inflammation and autophagy, also participates in the regulation of the canonical pathway in SA-AKI. During sepsis, overactivated NF-κB can inhibit autophagy, whereas inhibition of NF-κB restores autophagic levels and alleviates renal injury (Feng et al., 2020; Pan et al., 2021; Yu et al., 2022). This mechanism indicates that the function of the canonical autophagy pathway in SA-AKI is not only governed by energy metabolism but also profoundly influenced by inflammatory signals.

### 4.2 Key molecules involved in autophagy in SA-AKI

The execution of autophagy depends on the coordinated action of a series of autophagy-related proteins (Atgs). In SA-AKI, abnormal expression or function of these proteins directly affects the smoothness of autophagic flux, thereby determining the fate of renal TECs. Key proteins that have been extensively studied include microtubule-associated protein one light chain 3 (LC3), the autophagy receptor p62, the autophagy initiator Beclin-1, and core Atg family members.

#### 4.2.1 Sirtuin (SIRT) family

The SIRT family consists of seven isoforms (SIRT1-SIRT7) that are NAD^+^-dependent histone deacetylases involved in inflammation, energy metabolism, and autophagy regulation (Deng Z. et al., 2021; Bai X. et al., 2022). In SA-AKI, SIRT1, SIRT3, and SIRT6 have been shown to exert protective effects (Deng Z. et al., 2021; Bai X. et al., 2022). Activation of SIRT1 induces deacetylation of Beclin-1, thereby initiating autophagy and alleviating SA-AKI (Deng Z. et al., 2021). SIRT3 is similarly involved in SA-AKI: in CLP-induced septic AKI, SIRT3 overexpression enhances autophagy, reduces tubular cell apoptosis, and decreases the accumulation of pro-inflammatory cytokines, effects mediated by the AMPK/mTOR pathway (Zhao W. et al., 2018). Melatonin promotes mitophagy through SIRT3-mediated deacetylation of mitochondrial transcription factor A (TFAM), thereby attenuating SA-AKI (Deng Z. et al., 2021). SIRT6 has been shown to regulate autophagy. Zhang et al. found that LPS upregulates SIRT6 expression in HK-2 cells; overexpression of SIRT6 inhibits apoptosis and promotes autophagy, whereas silencing of SIRT6 increases the secretion of TNF-α and IL-6, reduces autophagy, and exacerbates LPS-induced renal injury (Zhang Y. et al., 2019). These results indicate that SIRT family members are positive regulators of autophagy in SA-AKI.

#### 4.2.2 Calcium/calmodulin-dependent protein kinase (CaMK)

Calcium/calmodulin-dependent protein kinase (CaMK) has recently been implicated in SA-AKI. In a septic mouse model, CaMKIV was found to enhance autophagy in macrophages and the kidney by inhibiting the serine phosphorylation of glycogen synthase kinase-3β (GSK-3β) and preventing the recruitment of F-box and WD repeat domain-containing 7 (FBXW7), thereby blocking the ubiquitin-proteasome degradation of mTOR (Zhang et al., 2014). This finding challenges the conventional view that mTOR inhibition promotes autophagy, suggesting that CaMKIV may regulate autophagy through an mTOR-independent pathway. The study demonstrated that CaMKIV plays a critical role in autophagy regulation in LPS-induced AKI (Zhang et al., 2014).

#### 4.2.3 Beclin-1

Beclin-1 is the mammalian homolog of yeast Atg6 and plays a key role in autophagy regulation; its expression level reflects autophagic activity. Human Beclin-1 contains a BH3 homology domain, a central coiled-coil domain, and an evolutionarily conserved C-terminal domain (Xu and Zhou, 2023). In SA-AKI, multiple studies have confirmed the protective function of Beclin-1-mediated autophagy. Xu and Zhou reported that downregulation of the long non-coding RNA MIAT promotes Beclin-1-mediated autophagy activation by interacting with PTBP1, thereby alleviating LPS-induced inflammatory injury in HK-2 cells (Xu and Zhou, 2023). Liu et al. found that co-culture of LPS with HK-2 cells induced significant autophagy and increased Beclin-1 expression (Liu J.-X. et al., 2020). Deng et al. confirmed that resveratrol alleviates AKI in septic mice by enhancing Beclin-1 deacetylation-mediated autophagy (Deng Z. et al., 2021; Bai X. et al., 2022). Jia et al. discovered that α-lipoic acid improved renal function in SA-AKI mice by upregulating Atg5, Atg7, and Beclin-1 (Jia et al., 2019). Thus, seeking autophagy agonists that stimulate Beclin-1 represents a beneficial strategy to combat SA-AKI (Jia et al., 2019).

#### 4.2.4 Toll-like receptor (TLR) family

TLRs are pattern recognition receptors localized on the cell surface. They recognize PAMPs, play a central role in innate immunity, and participate in the regulation of autophagy during sepsis (Liu W. et al., 2023). Liu et al. reported that enhancing the expression of miR-342–5p in exosomes derived from adipose-derived mesenchymal stem cells (AMSCs) accelerates autophagy by inhibiting TLR9, thereby ameliorating AKI in septic mice (Liu Y. et al., 2023). Leventhal et al. isolated renal TECs from mice lacking functional TLR4 and incubated them with LPS; unlike control mice, no autophagy was induced in those TECs (Leventhal et al., 2016). Another study demonstrated that the TLR4 inhibitor TAK242 effectively alleviates LPS-induced septic AKI by downregulating TLR4 and thereby inhibiting autophagy (Li and Feng, 2022). These studies indicate a close relationship between autophagy and TLR signaling in SA-AKI, and suggest that TLR4 may be an essential upstream molecule for LPS-induced autophagy in renal TECs.

#### 4.2.5 Atg family members

Atg family members can be considered core executors of autophagosome assembly. Atg7 is an E1-like ligase responsible for activating LC3 and Atg12; Atg5 forms a complex with Atg12 and Atg16L1, participating in the extension of the autophagosome membrane. Deletion of these proteins completely blocks autophagy. Leventhal et al. used conditional Atg7-knockout mice and found that after LPS challenge, renal TECs lacking Atg7 exhibited more severe tubular injury, significantly elevated blood urea nitrogen, and increased expression of IL-6 and phosphorylated STAT3 in renal tissue (Leventhal et al., 2016). This directly demonstrates the protective role of Atg7-mediated autophagy in SA-AKI. Moreover, α-lipoic acid upregulates Atg5 and Atg7 expression and improves renal function in SA-AKI mice (Jia et al., 2019).

In summary, the mechanisms of autophagy in septic acute kidney injury exhibit a multi-pathway, multi-protein network. In terms of the canonical pathway, the mTOR/AMPK-ULK1-Beclin-1-LC3 axis constitutes the core axis for autophagy initiation and execution; its activation helps clear damaged organelles and suppress inflammatory responses. P62, as an autophagy substrate, reflects the smoothness of autophagic flux. Regarding key regulatory proteins, SIRT1/3/6 enhance autophagy through deacetylation or the AMPK/mTOR pathway; CaMKIV provides an mTOR-independent regulatory mechanism; Beclin-1 and Atg7 are indispensable molecules for autophagy initiation and execution; and TLR4 serves as an upstream receptor mediating LPS-induced autophagic signaling. However, autophagy in SA-AKI is not a purely protective factor, early activation favors cell survival, but excessive or blocked autophagy can exacerbate renal injury. This “double-edged sword” characteristic suggests that precisely regulating the level of autophagy, rather than simply activating or inhibiting it, is the core direction for future research. Based on the above mechanisms, the development of drugs targeting autophagy-related pathways and proteins is becoming a new strategy for SA-AKI therapy.

## 5 Ferroptosis in SA-AKI

Ferroptosis is an iron-dependent, novel form of regulated cell death distinct from apoptosis and autophagy, characterized by glutathione depletion and lethal lipid peroxide accumulation leading to plasma membrane rupture (Dolma et al., 2003). Since it was named in 2012, ferroptosis has been demonstrated to participate in the pathophysiology of multiple organs, including the nervous system, liver, heart, and kidneys (Xie et al., 2016; Fang et al., 2019; Gao et al., 2015). In recent years, the role of ferroptosis in sepsis and its related organ injuries has garnered increasing attention. In the septic state, systemic inflammation and oxidative stress can lead to iron overload, inhibition of glutathione peroxidase 4 (GPX4) activity, and accumulation of lipid peroxidation products in renal TECs, thereby triggering ferroptosis and exacerbating renal tissue injury (Su et al., 2019). However, ferroptosis is not a single pathogenic factor in sepsis. On the one hand, during the bacterial infection phase of severe pancreatitis, ferroptosis in intestinal epithelial cells can disrupt the intestinal barrier, promoting the translocation of pathogenic intestinal bacteria and toxins into the circulation and extraintestinal tissues (Ma et al., 2021); on the other hand, in the early stage of the immune response, iron and lipid peroxidation are dramatically increased in macrophages, and ferroptosis inducers can help immune cells kill bacteria, exerting a defensive function (Ma et al., 2022). This “double-edged sword” property suggests that the role of ferroptosis in sepsis should be analyzed according to cell type and pathological stage. Nevertheless, the prevailing view remains that ferroptosis-induced inflammatory dysregulation is a major driver of septic organ injury (Amaral et al., 2019; Matsushita et al., 2015). Luo et al. demonstrated in an LPS-induced septic kidney injury mouse model that dexmedetomidine inhibits ferroptosis through the Keap1-Nrf2/HO-1 pathway, thereby modulating oxidative stress and treating SA-AKI, indicating that ferroptosis is a potential therapeutic target for this disease (Luo R.-R. et al., 2024).

Ferroptosis is primarily driven through two classical defense pathways that are suppressed in SA-AKI: the cystine/glutamate antiporter System Xc^−^-GSH-GPX4 axis and the FSP1/CoQ10/NADPH pathway (Dixon et al., 2012; Bergmann et al., 2009). In SA-AKI, renal TECs face multiple insults including systemic inflammation, oxidative stress, and iron metabolism disorders. The functions of these two pathways are suppressed, leading to the accumulation of lipid peroxides, ultimately triggering ferroptosis and exacerbating renal injury (Dixon et al., 2012). Recent animal and cell studies have revealed the specific roles of these pathways in SA-AKI.

### 5.1 The System Xc^−^-gsh-gpx4 axis: the core regulatory pathway

System Xc^−^ is a cystine/glutamate antiporter composed of SLC7A11 and SLC3A2. Its physiological function is to import extracellular cystine into cells for the synthesis of GSH (Dixon et al., 2012; Bergmann et al., 2009). GSH serves as the reductive cofactor for GPX4. GPX4 is the only enzyme in the body that can reduce lipid hydroperoxides in biological membranes to non-toxic lipid alcohols, and its activity directly determines the sensitivity of cells to ferroptosis (Liu S. et al., 2022; Liu et al., 2021). GPX4 belongs to the glutathione peroxidase superfamily and contains a catalytic selenocysteine residue that is essential for its activity (Flohé et al., 2022; Xie et al., 2023). In SA-AKI, inflammatory cytokines can inhibit System Xc^−^ activity, reduce cystine uptake, limit GSH synthesis, decrease GPX4 activity, and promote the accumulation of lipid peroxides, thereby inducing ferroptosis (Liu Y. et al., 2022; Guo et al., 2024a). Zhang et al. found that in a CLP-induced septic mouse model, levels of the ferroptosis markers malondialdehyde (MDA) and 4-hydroxynonenal (4-HNE) were significantly elevated, while GSH levels and GPX4 activity were markedly reduced (Zhang Y. et al., 2024; Zhang J. et al., 2024; Xiao et al., 2021). Treatment with the ferroptosis inhibitor Fer-1, which scavenges lipid radicals or protects GPX4 activity, effectively ameliorated tubular injury and renal dysfunction (Xiao et al., 2021; Qiongyue et al., 2022). Furthermore, augmenter of liver regeneration (ALR) inhibited ferroptosis in renal ischemia-reperfusion injury by maintaining the function of the GSH-GPX4 system (Huang et al., 2019). Xiao et al. also showed that maresin conjugates in tissue regeneration-1 (MCTR1) upregulates GPX4 expression by activating Nrf2, thereby suppressing ferroptosis in LPS-induced HK-2 cells and the kidneys of CLP mice (Xiao et al., 2021). Zhou et al. identified that cationic transporter regulator 1 (CHAC1) is upregulated in HK-2 cells under septic conditions and promotes ferroptosis by downregulating GPX4 (Zhou and Zhang, 2023). Conversely, the RNA-binding protein p23 can directly bind to GPX4 mRNA and protein, forming a protective complex that inhibits ferroptosis (Luo et al., 2025). Collectively, the System Xc^−^-GSH-GPX4 axis acts as a “brake” in SA-AKI, and its dysfunction is a key switch for ferroptosis.

### 5.2 The FSP1/coq10/NADPH pathway: an inhibitory pathway independent of GPX4

Ferroptosis suppressor protein 1 (FSP1, also known as AIFM2) is another important ferroptosis inhibitory pathway discovered in recent years. FSP1 consumes NADPH to reduce coenzyme Q10 (CoQ10) to ubiquinol (CoQ10H2), which acts as a lipophilic antioxidant to capture lipid radicals, directly inhibiting the chain reaction of lipid peroxidation and thereby preventing ferroptosis (Guo et al., 2024a). This pathway acts synergistically with the GPX4 pathway, constituting a dual defense line against ferroptosis. In SA-AKI, the FSP1 pathway is also suppressed. Guo et al. found that ginsenoside Rg1 upregulates FSP1 expression, increases CoQ10 and CoQ10H2 levels, reduces the NADPH/NADP^+^ ratio, and decreases the accumulation of lipid peroxidation products, thereby inhibiting ferroptosis in LPS-induced HK-2 cells and alleviating SA-AKI (Guo et al., 2024a). In addition, Pontel et al. suggested that CpG islands in the promoter region of the FSP1 gene can be methylated, silencing FSP1 expression and promoting ferroptosis (Pontel et al., 2022). These findings indicate that activating the FSP1/CoQ10/NADPH pathway can effectively protect renal TECs and represents a promising target for SA-AKI therapy.

### 5.3 Iron metabolism and lipid peroxidation: upstream drivers of ferroptosis

Ferroptosis depends on intracellular Fe^2+^ overload. In the septic state, elevated IL-6 induces increased hepcidin production in the liver, leading to degradation of the iron exporter ferroportin in renal TECs and consequent intracellular iron retention (Yang et al., 2025; Zhu et al., 2022). Excessive Fe^2+^ generates hydroxyl radicals *via* the Fenton reaction (Fe^2+^ + H_2_O_2_ → Fe^3+^ + OH· + OH^−^), which attack polyunsaturated fatty acids (PUFAs) in cell membranes. These are then catalyzed by ACSL4 and LOX to produce toxic lipid peroxides, disrupting membrane integrity (Chen X. et al., 2021; Jiang et al., 2021; Henning et al., 2022). ACSL4 catalyzes the activation of PUFAs to PUFA-CoA, which are then incorporated into membrane phospholipids as primary substrates for peroxidation (Fan et al., 2025). Yang et al. confirmed that inhibiting ACSL4 reduces ferroptosis under various conditions (Jiang et al., 2021). In SA-AKI animal models, the iron chelator deferoxamine (DFO) reduces free iron levels, attenuates lipid peroxidation, and alleviates renal injury (Liu Y. et al., 2022; Mitsuishi et al., 2012). Moreover, Xiao et al. found that the circadian gene NFIL3 is highly expressed in sepsis and promotes tubular ferroptosis by upregulating ACSL4; knockdown of NFIL3 reduced ferroptosis and the inflammatory response (Xiao et al., 2024).

### 5.4 The nrf2/HO-1 pathway: the intersection of antioxidant defense and ferroptosis

Nrf2 is a core transcription factor in the antioxidant response, upregulating the expression of SLC7A11 and GPX4. In SA-AKI, the expression and activity of SIRT1 are suppressed, leading to insufficient activation of the Nrf2 pathway and increased susceptibility to ferroptosis (Qiongyue et al., 2022). Qiong et al. found that irisin promotes Nrf2 deacetylation and nuclear translocation by activating SIRT1, thereby upregulating HO-1 and GPX4 expression, reducing ROS production and lipid peroxidation, and ameliorating SA-AKI (Qiongyue et al., 2022). Luo et al. also demonstrated that dexmedetomidine inhibits ferroptosis *via* the Keap1-Nrf2/HO-1 pathway, modulating oxidative stress and treating SA-AKI (Luo X. et al., 2024). Additionally, melatonin suppresses ferroptosis in SA-AKI by activating the Nrf2/HO-1 signaling pathway (Qiu et al., 2022). These studies suggest that the Nrf2/HO-1 pathway is a key node linking antioxidant defense and ferroptosis inhibition.

### 5.5 Ferroptosis-inflammation amplification loop in SA-AKI

In SA-AKI, the functions of the System Xc^−^-GSH-GPX4 axis and the FSP1/CoQ10/NADPH pathway are suppressed, while iron metabolism disorders lead to Fe^2+^ overload and enhanced lipid peroxidation. Together, these three factors drive ferroptosis in renal TECs. Rupture of ferroptotic cells releases DAMPs such as HMGB1 and SAP130, which activate the TLR4/MyD88/NF-κB pathway in macrophages, promoting the secretion of inflammatory cytokines including TNF-α and IL-6, thus forming a “ferroptosis-inflammation amplification loop” (Zhang J. et al., 2024; Ganz and Nemeth, 2015; Sun et al., 2020). Zhang et al. confirmed that SAP130 released from ferroptotic renal TECs can bind to the Mincle receptor on macrophages, promoting M1 macrophage polarization and further exacerbating ferroptosis (Zhang J. et al., 2024). Therefore, these classical pathways act as “protective barriers” in SA-AKI, and their inactivation is a key step in the occurrence of ferroptosis and the aggravation of renal injury. Activating these pathways (e.g., with Fer-1, ginsenoside Rg1, DFO, dexmedetomidine, *etc.*) can inhibit ferroptosis, making them potential therapeutic strategies for SA-AKI (Guo et al., 2024a; Qiongyue et al., 2022; Luo X. et al., 2024).

## 6 Necroptosis in SA-AKI

Necroptosis is a tightly regulated form of programmed cell death characterized by specific morphological features, including loss of plasma membrane integrity, cytoplasmic vacuolization (hyalinization), mitochondrial and endoplasmic reticulum swelling, cell lysis, and the release of intracellular contents (Bertheloot et al., 2021). In contrast to apoptosis, necroptosis is markedly immunogenic. It activates the innate immune response and triggers a robust inflammatory cascade through the release of DAMPs, such as HMGB1 and ATP (Kaczmarek et al., 2013). Within the pathological process of sepsis, a moderate degree of necroptotic activation can serve as an innate immune defense mechanism, eliminating host cells infected by pathogens. However, excessive or dysregulated necroptosis precipitates a runaway systemic inflammatory response, disrupts vascular endothelial barrier integrity, and drives the development of multiple organ dysfunction syndrome (Opal and Van Der Poll, 2015; Weng et al., 2014). Current research has established necroptosis as one of the core molecular mechanisms underlying the occurrence of SA-AKI. This section will systematically elucidate the core molecular components and regulatory mechanisms of the canonical necroptotic pathway, along with the pathophysiological evidence for its mediation of SA-AKI, thereby providing a theoretical foundation for therapeutic strategies targeting necroptosis in SA-AKI.

The canonical necroptotic pathway is mediated by a cascade of three core molecules: receptor-interacting protein kinase 1 (RIPK1), RIPK3, and mixed lineage kinase domain-like protein (MLKL) (Morgan and Kim, 2025). This pathway is independent of caspase protease activation, with its pivotal events involving necrosome assembly and the translocation and membrane insertion of MLKL. In the context of SA-AKI, aberrant activation of this pathway directly drives necrosis of renal tubular epithelial cells. Concurrently, it amplifies the inflammatory response *via* DAMP release, establishing a self-perpetuating “cell death-inflammation activation” positive feedback loop that further exacerbates renal injury (Zhou et al., 2024). Transmission electron microscopy has confirmed that activated MLKL undergoes oligomerization and specifically localizes to the lysosomal membrane. This localization induces increased lysosomal membrane permeability, subsequently activating lysosome-dependent cell death pathways and amplifying tubular epithelial damage (Li Z.-L. et al., 2024; Liu et al., 2024). These mechanisms have been validated in renal biopsy specimens from SA-AKI patients, which typically exhibit classic features of tubular injury, including extensive sloughing of tubular epithelial cells, proteinaceous cast formation within tubular lumens, and progressive interstitial edema (Liu et al., 2024).

### 6.1 Receptor-interacting protein kinase 1: the key initiating molecule of the necroptotic pathway

RIPK1 is a serine/threonine kinase whose protein structure, from N- to C-terminus, comprises a kinase domain, a RIP homotypic interaction motif (RHIM) domain, and a death domain (DD) (Tang et al., 2019). Under resting conditions, RIPK1 activity is strictly regulated by post-translational modifications such as ubiquitination. Upon activation of death receptors like tumor necrosis factor receptor 1 (TNFR1) by their ligands, RIPK1 undergoes deubiquitination and site-specific phosphorylation (e.g., autophosphorylation at S161 in mice). Its RHIM domain then interacts with the RHIM domain of RIPK3, recruiting RIPK3 to form a complex (Meng et al., 2021; Zhang et al., 2017). Additionally, RHIM-containing proteins such as Z-DNA binding protein 1 (ZBP1) or the Toll-like receptor 3/4 (TLR3/4) adaptor TRIF (TIR domain-containing adapter-inducing interferon-β) can directly engage RIPK3 *via* their RHIM domains, thereby bypassing RIPK1 to activate the necroptotic pathway (Upton et al., 2012; Kaiser et al., 2013). In SA-AKI animal models, the RIPK1-specific inhibitor Nec-1significantly attenuates renal tubular epithelial necrosis, reduces serum creatinine levels, and improves animal survival (Pefanis et al., 2023). Clinical cohort studies demonstrate that RIPK1 expression levels in peripheral blood mononuclear cells from septic patients are positively correlated with sequential organ failure assessment (SOFA) scores and procalcitonin concentrations, suggesting its potential as a biomarker for assessing sepsis severity (Wang et al., 2017).

### 6.2 Receptor-interacting protein kinase 3: the core mediating kinase of the necroptotic pathway

RIPK3 serves as the central mediating kinase of the necroptotic pathway. Its structure, from N- to C-terminus, consists of a kinase domain and a RHIM domain (Yan et al., 2022). RIPK3 assembles the necrosome—the core molecular event in necroptosis—by engaging in RHIM-RHIM interactions with RIPK1 or ZBP1 (Ai et al., 2024). The kinase activity of RIPK3 is the sole upstream source for MLKL phosphorylation, and its levels of autophosphorylation and total expression positively correlate with the intensity of necroptotic activation (Reynoso et al., 2017). RIPK3 activity is precisely regulated by various post-translational modifications: E3 ubiquitin ligases such as CHIP and PELI1 mediate K48-linked ubiquitination of RIPK3, targeting it for proteasomal degradation and thus exerting negative regulatory control over necroptosis (Choi et al., 2018; Seo et al., 2016). Conversely, O-linked β-N-acetylglucosamine (O-GlcNAc) modification can inhibit necrosome assembly and necroptotic activation by disrupting the interaction between RIPK1 and RIPK3 (Li et al., 2019a). During the pathogenesis of SA-AKI, RIPK3 expression is significantly upregulated in renal tubular epithelial cells, with its levels positively correlating with the extent of tubular necrosis and the degree of serum creatinine elevation (Dara, 2018). Research by Sureshbabu et al. confirmed that RIPK3 contributes to SA-AKI development by inducing mitochondrial respiratory chain dysfunction, excessive reactive oxygen species production, and loss of mitochondrial membrane potential (Sureshbabu et al., 2018). Xue et al. demonstrated that intervention with the RIPK3-selective inhibitor GSK872 significantly lowered serum creatinine, reduced tubular necrosis area, and ameliorated renal microcirculatory disturbances in septic mice (Xue et al., 2022). Clinical data indicate that plasma RIPK3 levels are markedly higher in patients with severe sepsis and septic shock compared to those with uncomplicated sepsis. Furthermore, high RIPK3 expression is independently associated with the incidence of SA-AKI and elevated 30-day all-cause mortality (Shashaty et al., 2019).

### 6.3 Mixed lineage kinase domain-like protein: the core effector executioner of the necroptotic pathway

MLKL functions as the principal effector executioner of the necroptotic pathway. Its structure, from N- to C-terminus, contains a four-helix bundle (4 H B) domain and a pseudokinase domain (Reynoso et al., 2017). In its resting state, MLKL maintains an autoinhibitory conformation through intramolecular interactions. Upon phosphorylation by RIPK3 (at S345 in mice, T357/S358 in humans), MLKL undergoes a conformational change that exposes its N-terminal 4 H B domain, which mediates MLKL oligomerization (Sun et al., 2012; Murphy et al., 2013). Oligomerized MLKL then translocates to the plasma membrane, inserting into the phospholipid bilayer to form cation-selective pores. This leads to loss of membrane integrity, osmotic imbalance, and ultimately cell lysis (Samson et al., 2020). In SA-AKI, phosphorylated MLKL (p-MLKL) is highly expressed in renal tubular epithelial cells, with its levels showing a strong positive correlation with the area of tubular necrosis and serum creatinine concentration (Jin et al., 2024). The MLKL-specific inhibitor necrosulfonamide (NSA) mitigates LPS-induced septic renal injury by blocking MLKL oligomerization (Bai et al., 2024; Adameova et al., 2022). Furthermore, research has revealed that MLKL can also translocate to the lysosomal membrane, inducing lysosomal membrane permeabilization and activating lysosome-dependent cell death pathways, thereby further amplifying tubular epithelial injury (Liu et al., 2024). Clinical studies have shown that MLKL and RIPK3 expression levels in renal tissue and peripheral blood cells from surviving septic patients are significantly higher than in non-survivors, suggesting that MLKL expression may be independently associated with prognosis in septic patients (Mallarpu et al., 2021).

#### 6.3.1 Post-translational regulation of necroptotic signaling

Beyond the core kinase cascade, the activity of RIPK1 and MLKL is further modulated by post-translational modifications that fine-tune the threshold of necroptotic activation. For RIPK1, its deubiquitination by CYLD removes inhibitory ubiquitin chains and facilitates necrosome assembly, while phosphorylation by IKK2 at specific residues can limit its pro-death function (Li Z.-L. et al., 2024). RIPK1 autophosphorylation at S161 is required for RIPK3 recruitment, as discussed above (Liu et al., 2024). For MLKL, its oligomerization and membrane translocation are negatively regulated by thioredoxin-1, which maintains MLKL in a reduced state to suppress disulfide bond-dependent polymer formation (Yan et al., 2022). Notably, RIPK3 is also subject to extensive post-translational regulation—including ubiquitination by CHIP/PELI1 and O-GlcNAc modification, as detailed in Section 5.2 (Ai et al., 2024; Reynoso et al., 2017; Choi et al., 2018). These regulatory layers collectively determine whether death signals culminate in necroptotic execution or are halted at intermediate steps. The balance of these modifications is disrupted during sepsis, likely due to oxidative stress and metabolic derangements that affect ubiquitin-proteasome system activity and kinase-phosphatase equilibrium (Su et al., 2023; Li et al., 2019b).

### 6.4 Cascading amplification, pathological roles, and molecular crosstalk of the canonical necroptotic pathway

The aforementioned RIPK1-RIPK3-MLKL signaling axis constitutes the core cascade of the canonical necroptotic pathway, playing a central driving role in the pathogenesis of SA-AKI: RIPK1, as the pathway initiator, senses damage signals from death receptors or Toll-like receptors and recruits/activates RIPK3 *via* RHIM domain interactions; activated RIPK3, through its kinase activity, phosphorylates downstream MLKL; phosphorylated MLKL oligomerizes and inserts into the plasma membrane to form pores, culminating in cell lysis and DAMP release (Wu et al., 2021). Quantitative studies in a CLP-induced septic mouse model of SA-AKI estimate the contribution of this pathway to renal injury at approximately 68% ± 5%. Intervention with the RIPK3 inhibitor GSK872 reduced serum creatinine by 39% and improved 28-day survival by 22% (Fu et al., 2024). Moreover, there is clear molecular crosstalk between the necroptotic and pyroptotic pathways: DAMPs released by necroptosis can activate the NLRP3 inflammasome, promoting the cleavage of the pyroptosis executor GSDMD and the formation of pyroptotic pores. The two processes synergistically exacerbate renal tissue damage (Stark and Massberg, 2021; Pandey et al., 2025). In summary, targeting any of the core molecules, including RIPK1, RIPK3, or MLKL, can effectively block necroptotic pathway activation and mitigate pathological damage in SA-AKI, holding significant clinical translational potential.

## 7 Interplay of regulated cell death pathways in SA-AKI

In the pathological progression of SA-AKI, apoptosis, pyroptosis, necroptosis, autophagy, and ferroptosis do not occur in isolation. Instead, they form a complex RCD network through shared upstream signals, cross-regulation at key nodes, and synergistic effects. A thorough understanding of the crosstalk among these death modalities lies at the core of pathophysiological research on SA-AKI and provides the theoretical foundation for developing multi-target combination therapies.

### 7.1 Caspase-8: the molecular switch regulating apoptosis, pyroptosis, and necroptosis

Caspase-8 is the initiator caspase of the extrinsic apoptotic pathway, and also a key node that regulates the switching between multiple RCD modalities. In SA-AKI, the activity status of caspase-8 directly determines the mode of cell death: when caspase-8 activity is normal, cells undergo apoptosis; when caspase-8 is inhibited or deficient, death signals shift to the necroptosis pathway, executing programmed necrosis *via* the RIPK1-RIPK3-MLKL signaling axis. More importantly, studies by Fritsch et al. demonstrated that the embryonic lethal phenotype of *caspase-8* knockout mice can be rescued by knockout of *Ripk3* or *Mlkl*, while simultaneous knockout of *Ripk3*, *Mlkl*, and *Gsdmd* can completely prevent embryonic lethality (Fritsch et al., 2019). In SA-AKI, the activity status of caspase-8 directly determines the formation of the RIPK1/RIPK3/MLKL necrosome. Wu et al. pointed out that caspase-8 initiates the caspase-8-driven apoptotic pathway during apoptosis, while the RIPK1-caspase-8 complex also participates in mediating necroptosis (Wu Z. et al., 2022). Under the septic microenvironment, elevated cFLIP expression inhibits the activation of caspase-8 in the death receptor complex, thereby suppressing its anti-necroptotic function, allowing the RIPK1/RIPK3/MLKL signaling pathway to be activated (Marini et al., 2015). The activation or deficiency of caspase-8 enzymatic activity is closely associated with PANoptosis, so caspase-8 is defined as a key regulator of cell survival or death (Zhang W. et al., 2024). In SA-AKI renal tissue, PANoptosis-related molecules including RIPK1, MLKL, caspase-3/7, and FADD are all significantly upregulated, and ZBP1 is markedly elevated in the renal interstitium; these findings reveal the critical role of ZBP1-driven PANoptosis in the pathogenesis of SA-AKI (Chowdhury and Hasan, 2025). Therefore, caspase-8 is not only the initiator caspase of extrinsic apoptosis, but also the molecular switch that determines whether cells undergo apoptosis or pyroptosis/necroptosis, and its activity status directly alters the death fate of renal tubular epithelial cells in SA-AKI.

### 7.2 Synergistic effects of GSDMD and MLKL: co-executors of pyroptosis and necroptosis

GSDMD-mediated pyroptosis and MLKL-mediated necroptosis are two important inflammatory RCD modalities in SA-AKI. The two not only act independently, but can also synergistically exacerbate renal tissue injury *via* shared regulatory nodes. Chen et al. confirmed in a CLP-induced polymicrobial sepsis mouse model that simultaneous blockade of necroptosis and pyroptosis (i.e., dual knockout of RIPK3/GSDMD or MLKL/GSDMD) produces a cumulative protective effect, significantly alleviating septic shock, systemic coagulopathy, and multi-organ injury (Chen et al., 2020). This study further confirmed *via* bone marrow transplantation experiments that both necroptosis and pyroptosis occurring in myeloid and non-myeloid cells play indispensable roles in the progression of sepsis-associated multi-organ injury (Chen et al., 2020). Additionally, Chen et al. found that inflammatory cytokine and HMGB1-induced cell death can also be blocked by dual knockout of RIPK3/GSDMD or MLKL/GSDMD, suggesting the existence of a positive feedback loop of inflammatory amplification between the RIPK3/MLKL and GSDMD pathways (Chen et al., 2020). Although the above evidence comes from a systemic sepsis model, interactions between the two death modalities have also been observed in kidney-specific injury models. Tonnus et al. reported that in ischemia-reperfusion injury and cisplatin-induced acute kidney injury, GSDMD-deficient mice exhibited more severe renal tubular injury and significantly elevated serum urea and creatinine levels compared to wild-type control mice; notably, this hypersensitivity was reversed by combined knockout of GSDMD and MLKL (Tonnus et al., 2022). This indicates that GSDMD plays a non-cell-autonomous role in protecting renal tubules from necroptosis-mediated injury. The above evidence demonstrates that GSDMD and MLKL are not simply parallel pathways in SA-AKI, but form a synergistic amplified death system *via* the shared RIPK3 regulatory node and plasma membrane pore-forming mechanisms. Combined targeting of RIPK3/GSDMD or MLKL/GSDMD has shown a cumulative protective effect in sepsis animal models, suggesting that simultaneous blockade of pyroptosis and necroptosis may be a potential therapeutic strategy for SA-AKI.

### 7.3 Dynamic balance between autophagy and apoptosis/necroptosis

Autophagy in SA-AKI is not simply protective or damaging, but a dynamically regulated process. Wu et al. pointed out that in the early stage of sepsis-associated AKI, autophagosomes form and inhibit various forms of regulated cell death; as the disease progresses, regulated cell death is gradually initiated (Wu Z. et al., 2022). In renal tubular epithelial cells (HK2 cells), the expression of autophagy-related proteins LC3-II and Beclin one changes in a time-dependent manner after LPS stimulation: they are significantly upregulated at early time points (2–8 h), and gradually decline at late stages (16–24 h); a similar temporal change pattern of autophagy proteins is also observed in renal tubular epithelial cells after CLP surgery (Dai et al., 2019). This temporal characteristic is negatively correlated with the changes in apoptosis/pyroptosis markers, providing direct temporal evidence from both cellular and animal models for the notion that “caspase-3-mediated apoptosis requires inhibition of autophagy to be fully executed”. Knockout of the TREM1 gene significantly alleviates CLP-induced SA-AKI renal injury: manifested as reduced renal tubular injury scores, improved serum creatinine and blood urea nitrogen levels, and decreased circulating cytokines; pyroptosis markers (NLRP3, cleaved caspase-1, cleaved GSDMD, IL-1β) in renal tissue are all significantly downregulated (Liu et al., 2026). At the autophagy level, autophagy markers LC3B, Beclin 1, and Atg5 are all elevated in *Trem1* knockout mice. In LPS-stimulated HK2 cells, TREM1 overexpression significantly increases NLRP3 activation, IL-1β release, and pyroptosis markers, while TREM1 silencing produces the opposite phenotype (Liu et al., 2026). This result indicates that in SA-AKI, when TREM1-NLRP3-mediated pyroptosis is inhibited, autophagy activity shows an increasing trend; conversely, when pyroptosis is excessively activated, autophagy is impaired. In summary, autophagy acts as the “gatekeeper” of RCD in SA-AKI: moderate autophagy activation in the early stage of sepsis inhibits various forms of RCD and delays the progression of renal injury; while autophagy dysfunction or depletion leads to successive loss of control over apoptosis, pyroptosis, and necroptosis, accelerating the deterioration of renal function.

### 7.4 Cross-regulation between autophagy and ferroptosis: ferritinophagy

Autophagy can regulate ferroptosis *via* selective autophagy forms. Pan et al. revealed selective autophagy (e.g., ferritinophagy) affects the occurrence of ferroptosis; autophagy deficiency in AKI exacerbates renal tubular injury, while promoting autophagy has become an important therapeutic strategy (Pan et al., 2024). Ferritinophagy is mediated by nuclear receptor coactivator 4 (NCOA4), which transports ferritin to autolysosomes for degradation, releasing free Fe^2+^, which catalyzes lipid peroxidation *via* the Fenton reaction and triggers ferroptosis (Zhang P. et al., 2024). In SA-AKI, the NCOA4-ferritin heavy chain 1 (FTH1)-mediated ferritinophagy has been confirmed as a key therapeutic target for sepsis-induced organ injury (Zhang P. et al., 2024). Wu et al. confirmed *via* single-cell RNA sequencing, genetic, and mass spectrometry techniques that STING directly interacts with the coiled-coil domain of NCOA4 *via* its CBD domain, triggering ferritinophagy-mediated ferroptosis, while maintaining STING dimer stability to enhance inflammatory responses; inhibition of STING-NCOA4 interaction can reduce ferroptosis in peripheral blood monocytes of sepsis patients and lower the mortality of sepsis mice (Wu J. et al., 2022). In sepsis-induced AKI renal tubules, STING expression is upregulated and promotes NCOA4-mediated ferritinophagy and ferroptosis (Jin L. et al., 2023). Additionally, HMGB1 plays an important role in SA-AKI ferroptosis. In an LPS-induced AKI mouse model, HMGB1 expression in renal tissue is elevated; knockdown of HMGB1 in podocytes alleviates injury; after HK2 cells are exposed to HMGB1, mitochondrial damage and apoptosis are significantly increased (Gao et al., 2021). Cytoplasmic translocation of HMGB1 can trigger ferroptosis *via* interaction with acyl-CoA synthetase long-chain family member 4 (ACSL4), amplifying lipid peroxidation and cell death^35. Inhibition of HMGB1 nucleocytoplasmic translocation or neutralization of its extracellular activity can alleviate inflammation and ferroptosis, thus relieving AKI (Li et al., 2025). The above mechanisms indicate that in SA-AKI, moderate mitophagy can remove damaged mitochondria, reduce mitochondrial reactive oxygen species (mtROS) and lipid peroxidation, thereby inhibiting ferroptosis; however, excessive ferritinophagy exacerbates ferroptosis by releasing free iron, forming a positive feedback injury amplification loop of “autophagy-ferroptosis”.

It should be noted, however, that the relationship between ferritinophagy and ferroptosis in SA-AKI is not unidirectional. In LPS-induced SA-AKI models, inhibition of NCOA4-mediated ferritinophagy has been shown to attenuate renal tubular injury and reduce ferroptosis (Zhang P. et al., 2024; Jin L. et al., 2023). However, the functional outcome of ferritinophagy likely depends on the degree of iron overload and the availability of antioxidant defenses. In the specific context of SA-AKI, systemic inflammation and oxidative stress can lead to iron overload in renal TECs (Su et al., 2019; Liu Y. et al., 2022). Under these conditions, ferritinophagy-mediated iron release may exacerbate lipid peroxidation and ferroptosis. Conversely, in settings where iron availability is limited, ferritinophagy may serve to maintain cellular iron homeostasis (Ganz and Nemeth, 2015). This duality has not been systematically examined in SA-AKI models, and whether ferritinophagy is predominantly protective or detrimental in SA-AKI likely depends on the severity of iron overload, the extent of GPX4 inactivation, and the cellular context. This represents an important direction for future investigation in SA-AKI.

### 7.5 Shared upstream signals: PAMPs/DAMPs, TLR4, and oxidative stress

The underlying reason for the simultaneous activation of multiple RCD modalities in SA-AKI is that they share multiple upstream danger signals and signal transduction pathways. TLR4 recognizes LPS and activates NF-κB *via* the MyD88-dependent pathway, releasing inflammatory mediators and amplifying local inflammatory responses. Clemastine pretreatment can reduce CLP-induced SA-AKI renal injury in rats by downregulating TLR4 and MYD88 expression and inhibiting phosphorylation of NF-κB p65 (Abdelnaser et al., 2025). TAK242 (a specific TLR4 inhibitor) can significantly improve renal function in SA-AKI rat models by inhibiting the TLR4/NF-κB pathway (Xia et al., 2024). Sepsis-induced ROS and reactive nitrogen species (RNS) reduce NO bioavailability, mediate renal microcirculation disorders, tissue hypoxia, and mitochondrial dysfunction, initiating a vicious cycle of cellular injury. MtROS and oxidized mtDNA produced by mitochondrial dysfunction serve as important DAMPs, further enhancing NLRP3 inflammasome activation and pyroptosis responses. In an LPS-induced SA-AKI mouse model, LPS injection activates the cGAS-STING axis and NLRP3 inflammasome, manifested as elevated phosphorylation levels of TBK1, IRF3, and NF-κB, increased cleaved caspase-1/caspase-1 ratio and GSDMD N-terminal/GSDMD ratio, and upregulated IL-1β and IL-18 levels; the cGAS inhibitor RU.521 can alleviate NLRP3 inflammasome activation and SA-AKI, while the STING agonist DMXAA can counteract this protective effect (Luo X. et al., 2024). In LPS-treated HK2 cells, cytoplasmic mtDNA release and activation of the cGAS-STING-NLRP3 axis are observed; inhibition of mtDNA replication reduces cytoplasmic mtDNA accumulation and downregulates this axis, improving LPS-induced cytotoxicity (Luo X. et al., 2024). The mitochondria-targeted antioxidant MitoTEMPO reduces mtROS production induced by sepsis serum, maintains mitochondrial membrane potential, decreases IL-1β levels, and improves renal function (Arulkumaran et al., 2021). The above evidence confirms the critical role of the mtROS-mtDNA-NLRP3 signaling axis in SA-AKI, forming a bidirectional amplification cycle between mitochondrial injury and RCD, and explaining the molecular basis for the simultaneous occurrence of multiple RCD modalities in SA-AKI.

## 8 Therapy

The extensive crosstalk among RCD pathways discussed in the preceding sections—particularly the caspase-8-dependent switch among apoptosis, pyroptosis, and necroptosis, the synergistic amplification of tissue damage by GSDMD and MLKL, and the autophagy-ferroptosis axis mediated by ferritinophagy, provides a mechanistic foundation for therapeutic intervention. Targeting individual RCD pathways has shown promise in preclinical studies, but the interconnected nature of these pathways also raises the possibility that multi-targeted strategies may achieve superior efficacy by simultaneously blocking multiple death modalities. In this section, we first summarize representative agents targeting each individual RCD pathway, then discuss the theoretical basis and current evidence for multi-targeted combination strategies, and finally address the translational challenges that must be overcome for clinical implementation.

### 8.1 Single-pathway targeting agents

Preclinical studies have demonstrated that pharmacological or genetic interventions modulating apoptosis, pyroptosis, necroptosis, autophagy, or ferroptosis can attenuate renal injury in experimental models of SA-AKI. Table 1 summarizes representative agents targeting each RCD pathway, their mechanisms of action, experimental models, and current stage of development.

**TABLE 1:** Therapeutic strategies based on RCD in SA-AKI.

RCDs	Agent	Mechanism/Target	Stage/Status	Effect in sa-aki	References
Apoptosis	Capsaicin	↓ caspase-8	Preclinical	↓Apoptosis	Bircan et al. (2025)
	Resveratrol	↓ iNOS, ↑ Bcl-2/Bcl-xL	Preclinical	↓Apoptosis	Chen et al. (2015)
	BAM15	↓ mtROS	Preclinical	↓Apoptosis	Tsuj et al. (2023)
	Flavonoid fisetin	↓ Src/NF-κB/MAPK	Preclinical	↓Apoptosis	Ren et al. (2020)
	Byakangelicin	↓ NF-κB pathway	Preclinical	↓Apoptosis	Hangda et al. (2024)
	Spermidine	↓ TLR4/MyD88/NF-κB	Preclinical	↓Apoptosis	Shen et al. (2025)
	Liensinine	↑ JNK/p38-ATF2 axis	Preclinical	↓Apoptosis	Zhang L. et al. (2023)
	Astragaloside IV (AS-IV)	Restores cleaved caspase-3 pathway	Preclinical	↓Apoptosis	Feng et al. (2022)
Pyroptosis	Immune Response Gene-1 (IRG1)/itaconate	↓ GSDMD	Preclinical	↓ Pyroptosis	Yang N. et al. (2024)
	tissue inhibitor of metalloproteinases 2 (TIMP2)	↓ caspase-1/NLRP3/GSDMD	Preclinical	↓Pyroptosis	Xu et al. (2024)
	chlorogenic acid (CGA)	↓ NLRP3 inflammasome	Preclinical	↓Pyroptosis	Fang et al. (2024)
	P. cuspidatum extracts (PCE)	↓ NF-κB	Preclinical	↓Pyroptosis	Yang Y. et al. (2024)
	VX-765	↓ caspase-1	Phase II (discontinued)	↓Pyroptosis	Wen et al. (2022)
	Maresin-1	↓ caspase-1/NLRP3/GSDMD	Preclinical	↓Pyroptosis	Sun M. et al. (2024)
	Alamandine (ALA)	↓ caspase-1	Preclinical	↓Pyroptosis	Songür et al. (2023)
	Erbin	↓ NLRP3 inflammasome	Preclinical	↓Pyroptosis	Liu W. et al. (2023)
	Theaflavin	↓ NLRP3 inflammasome	Preclinical	↓Pyroptosis	Chen S.-Y. et al. (2023)
	saroglitazar (SAR)	↓ caspase-11, GSDMD	Approved (dyslipidemia)	↓Pyroptosis	Francis et al. (2023)
	Wild-Type p53-Induced Phosphatase 1 (WIP1/PPM1D)	↓ p38 MAPK	Preclinical	↓Pyroptosis	Wang et al. (2024)
	Zn2+	↓ NLRP3 inflammasome	Preclinical	↓Pyroptosis	Guo et al. (2024b)
	Thymoquinone	↓ NLRP3, caspase-1, caspase-3	Preclinical	↓Pyroptosis	Guo et al. (2020)
	Micheliolide	↓ NLRP3 inflammasome	Preclinical	↓Pyroptosis	Lei et al. (2024)
	Protein Kinase R Inhibitor C16	↓ ASC, NLRP3, caspase-1	Preclinical	↓Pyroptosis	Zhou et al. (2020)
	Mdivi-1	↓ NLRP3 inflammasome	Preclinical	↓Pyroptosis	Liu R. et al. (2020)
	Compound 4–155	↓ RIPK1, RIPK3, MLKL	Preclinical	↓Pyroptosis	Ling et al. (2023)
Autophagy	procyanidin B2	↑ Nrf2 nuclear translocation	Preclinical	↑ Autophagy	Liu J.-X. et al. (2020)
	Zn2+	↑ SIRT7	Preclinical	↑ Autophagy	Guo et al. (2024b)
	FTO	↓ SNHG14/miR-373–3p/ATG7	Preclinical	↑ Autophagy	Yang Y. et al. (2024)
	Micheliolide	↑ Nrf2/PINK1/Parkin	Preclinical	↑ Autophagy	Lei et al. (2024)
	Melatonin	↑ SIRT3-mediated TFAM deacetylation	Preclinical	↑ Autophagy	Deng et al. (2024)
	Alcohol dehydrogenase 1	↑ PINK1-Parkin	Preclinical	↑ Autophagy	Zheng et al. (2023b)
	Resveratrol	↑ Beclin-1	Preclinical	↑ Autophagy	Deng Z. et al. (2021)
​	Ulinastatin	↓ LC3II	Approved (pancreatitis)	↑ Autophagy	Li et al. (2022b)
	Ascorbate	↑ SVCT-1/-2	Preclinical	↑ Autophagy	Chen Z.-D. et al. (2021)
	Rapamycin	↑ Beclin-1, ↑ LC3II/LC3I ratio	Approved (immunosuppressant)	↑ Autophagy	Li X. et al. (2024)
	Dexmedetomidine	↑ α2-AR/AMPK/mTOR	Approved (sedative)	↑ Autophagy	Yang et al. (2020)
Ferroptosis	Andrographolide	↑ SLC7A11, GPX4	Preclinical	↓ Ferroptosis	Zhang Y. et al. (2024)
	Klotho	↑ Nrf2	Preclinical	↓ Ferroptosis	Zhou et al. (2023)
	Maresin conjugates in tissue regeneration 1 (MCTR1)	↑ Nrf2	Preclinical	↓ Ferroptosis	Xiao et al. (2021)
	Ginsenoside Rg1	↑ FSP1, GPX4, GSH	Preclinical	↓ Ferroptosis	Guo et al. (2024a)
	Melittin	↑ GPX4	Preclinical	↓ Ferroptosis	Zan et al. (2024)
	Dexmedetomidine	↑ GPX4	Approved (sedative)	↓ Ferroptosis	Li et al. (2023)
	GYY4137	↑ GPX4	Preclinical	↓ Ferroptosis	Zhang W. et al. (2023)
	Fer-1 (Ferrostatin-1)	↓ lipid peroxidation	Preclinical	↓ Ferroptosis	Wang et al. (2025)
	Irisin	↑ SIRT1/Nrf2	Preclinical	↓ Ferroptosis	Qiongyue et al. (2022)
	Melatonin	↑ Nrf2/HO-1	Preclinical	↓ Ferroptosis	Qiu et al. (2022)
Necroptosis	Dexmedetomidine	↓ EMT	Approved (sedative)	↓ Necroptosis	Sun Q. et al. (2024)
	Nec-1 (Necrostatin-1)	↓ RIPK1	Preclinical	↓ Necroptosis	Pefanis et al. (2023)
	GSK872	↓ RIPK3	Preclinical	↓ Necroptosis	Xue et al. (2022)
	Necrosulfonamide (NSA)	↓ MLKL	Preclinical	↓ Necroptosis	Bai et al. (2024)
	4-HPA	↑ ARC, ↑ ARC-RIPK1 interaction	Preclinical	↓ Necroptosis	An et al. (2024)
Multi-target/Others	Astragalus polysaccharide (AP)	↓ RIPK1/RIPK3/MLKL ↑ autophagic flux	Preclinical	↓ Necroptosis ↑ Autophagy	Wang et al. (2026)
	Celastrol	↓ PKM2	Preclinical	↓ Inflammation, Warburg effect	Lu et al. (2022)

### 8.2 Rationale for multi-targeted combination strategies

The crosstalk mechanisms detailed in Section 6 provide a theoretical basis for combining agents that target different RCD pathways. Several rational combination strategies can be envisioned based on the interconnected nature of these death modalities.

First, simultaneous inhibition of caspase-8-dependent apoptosis and RIPK3/MLKL-mediated necroptosis may be particularly effective, given that caspase-8 functions as a molecular switch between these two pathways. When caspase-8 activity is compromised, death signals shift toward necroptosis (Wu Z. et al., 2022; Fritsch et al., 2019). Therefore, combining a caspase-8 inhibitor with a RIPK3 or MLKL inhibitor could theoretically block both apoptotic and necroptotic routes of cell death, preventing the compensatory activation that might occur with single-agent treatment.

Second, co-targeting GSDMD (pyroptosis) and MLKL (necroptosis) is supported by evidence that dual knockout of these effectors produces cumulative protection in sepsis models (Chen et al., 2020). Both GSDMD and MLKL form membrane pores that trigger inflammatory cell death, and their simultaneous blockade may more effectively limit tissue damage and DAMP release than targeting either pathway alone.

Third, the autophagy-ferroptosis axis offers another combination opportunity. Moderate enhancement of mitophagy can clear damaged mitochondria and reduce mtROS production, thereby inhibiting ferroptosis (Pan et al., 2024). However, excessive ferritinophagy (autophagic degradation of ferritin) releases free iron and promotes ferroptosis^212 213^. Thus, a strategy that enhances general autophagy while specifically inhibiting ferritinophagy, for example, by targeting NCOA4, could theoretically break the ferroptosis amplification loop. Agents such as melatonin and micheliolide, which promote mitophagy while reducing oxidative stress, may already exert such dual effects (Deng et al., 2024; Lei et al., 2024).

Fourth, shared upstream signaling nodes such as TLR4 and NF-κB represent additional targets for simultaneous modulation of multiple RCD pathways. TLR4 inhibition with TAK-242 has been shown to reduce both pyroptosis and apoptosis in SA-AKI models (Xia et al., 2024), and combining TLR4 blockade with pathway-specific inhibitors may yield additive or synergistic effects.

### 8.3 Translational challenges and future directions

Despite the extensive preclinical evidence summarized above, the clinical translation of RCD-targeting therapies for SA-AKI faces several significant challenges that merit careful consideration.

First, most agents remain at the preclinical stage. With the exception of a few agents already approved for other indications (rapamycin, dexmedetomidine, saroglitazar, ulinastatin) and one that has entered clinical trials for related conditions (VX-765, a caspase-1 inhibitor evaluated in Phase II trials but not advanced further for AKI), the majority of compounds listed in this table have only been tested in rodent models. Their safety, pharmacokinetics, and efficacy in humans remain unproven.

Second, target specificity and off-target effects remain major concerns. The challenges of translating RCD-targeting strategies from bench to bedside are perhaps best illustrated by the history of caspase inhibitors in sepsis. Although it shows promising preclinical efficacy in rodent models, the therapeutic effect of the broad-spectrum caspase inhibitor Z-VAD-FMK in patients with sepsis still needs to be confirmed (Li et al., 2019b). The main reasons might be immunosuppression and the increased risk of secondary infections. More recently, the hepatobiliary toxicity observed with the RIPK1 inhibitor DNL104 in a Phase I trial serves as a cautionary example. However, this toxicity is considered to be compound-specific (off-target) rather than a class effect, as other RIPK1 inhibitors such as GSK2982772 have not shown similar liver safety signals in clinical studies (Grievink et al., 2020). This underscores the challenges in drug development where pathway redundancy or compound-specific liabilities can lead to unexpected toxicities, potentially disrupting physiological cell turnover and immune homeostasis. These examples highlight a critical lesson: potent target engagement and robust efficacy in animal models do not guarantee clinical success, and the design of clinical trials for RCD inhibitors must account for the complex, dynamic nature of the host response in sepsis. The multi-targeted combinations proposed above, while theoretically attractive, carry even greater risks of overlapping toxicities and immune suppression that must be carefully evaluated.

Third, the timing of intervention is inherently difficult in sepsis. RCD pathways exhibit dynamic activation patterns during sepsis progression: autophagy is activated early but may become impaired later, while pyroptosis and necroptosis dominate in the inflammatory phase (Wu Z. et al., 2022; Dai et al., 2019). Administering an inhibitor too early might block beneficial adaptive responses, while intervention too late may miss the therapeutic window. This temporal complexity complicates clinical trial design and patient selection, and is particularly challenging for multi-targeted regimens that may require precise timing of each component.

Fourth, reliable biomarkers for patient stratification are lacking. Plasma levels of RIPK3, GSDMD fragments, LC3-II, and ferritin have been proposed as potential biomarkers of RCD activation^183,193,200^, but none have been validated for clinical use in SA-AKI. Without biomarkers to identify which RCD pathway is predominantly active in a given patient at a given time, targeted therapy, whether single-agent or multi-targeted, remains empirical rather than precision-based. The development of companion diagnostics should therefore proceed in parallel with therapeutic development. Furthermore, transcriptomic endotyping has emerged as a complementary approach: a validated whole-blood gene expression classifier has identified three sepsis endotypes, including inflammopathic, adaptive, and coagulopathic, that exhibit distinct biomarker profiles and clinical outcomes (Tavris et al., 2025). Combining such endotyping with protein-based biomarkers (e.g., NGAL, suPAR, bioactive adrenomedullin) has been shown to enhance predictive accuracy for kidney replacement therapy or death, achieving AUC values of 0.80–0.85 (Tavris et al., 2025). These findings suggest that a multi-parametric approach combining RCD-specific markers with broader endotype classification may eventually enable precision-based patient stratification. However, prospective validation in large, multicenter cohorts is urgently needed before any of these biomarkers can be integrated into clinical practice.

Fifth, drug delivery to the kidney presents unique anatomical and physiological barriers. The renal tubular epithelium is difficult to target with systemically administered agents due to the glomerular filtration barrier and tubular reabsorption mechanisms. Nanoparticle-based delivery strategies offer promise but remain at an early stage. For multi-targeted combination approaches, the challenge is magnified, as each agent may require different delivery characteristics.

Finally, the gap between preclinical models and human SA-AKI must be acknowledged. Most preclinical studies use LPS injection or CLP in young, healthy rodents. While these models recapitulate certain aspects of sepsis, such as systemic inflammation and acute tubular injury, they suffer from several critical limitations. First, LPS injection models a pure endotoxemic response that does not fully reflect the polymicrobial nature of most human sepsis. Second, CLP, while more clinically relevant, produces a relatively uniform and predictable disease course that does not capture the biological heterogeneity of human sepsis, which is influenced by diverse pathogens, host genetics, comorbidities, and prior treatments. Third, most rodent studies use young, healthy animals, whereas human SA-AKI typically occurs in elderly patients with pre-existing conditions such as chronic kidney disease, diabetes, or cardiovascular disease (Peerapornratana et al., 2019; Ronco et al., 2019). Fourth, the timing of intervention in preclinical studies is often optimized, drugs are administered prophylactically or at the earliest signs of injury, whereas in clinical practice, patients present with established disease, narrowing the therapeutic window. To improve translational success, future preclinical studies should consider aged or comorbidity-bearing animal models, delayed intervention paradigms, and patient-derived endotype-stratified approaches.

## 9 Conclusion

SA-AKI involves a complex network of RCD pathways, including apoptosis, pyroptosis, necroptosis, autophagy, and ferroptosis. These pathways are not independent; they are interconnected through shared upstream signals (PAMPs, DAMPs, TLR4, oxidative stress), key molecular switches (e.g., caspase-8), and synergistic effectors (e.g., GSDMD and MLKL). Autophagy acts as a dynamic gatekeeper, and ferritinophagy links autophagy to ferroptosis. Collectively, these mechanisms drive renal tubular injury and amplify inflammation. Targeting RCD pathways has emerged as a promising therapeutic strategy. Preclinical studies have shown that inhibitors of caspases, NLRP3, RIPK1/RIPK3/MLKL, autophagy regulators, and ferroptosis blockers can attenuate renal injury. Given the extensive crosstalk among RCD pathways, multi-targeted interventions (e.g., Astragalus polysaccharide) may offer synergistic benefits. Despite encouraging preclinical results, clinical translation faces challenges including drug specificity, safety, and identification of reliable biomarkers for patient stratification. Future research should focus on developing selective RCD inhibitors, kidney-targeted delivery systems, and combination therapies. Elucidating the dynamic interplay of RCD networks will pave the way for precision medicine in SA-AKI.
